# Quantifying dispersal of a non-aggressive saprophytic bark beetle

**DOI:** 10.1371/journal.pone.0174111

**Published:** 2017-04-13

**Authors:** Nicolas Meurisse, Stephen Pawson

**Affiliations:** 1 Scion, Rotorua, New Zealand; 2 Scion, Ilam, Christchurch, New Zealand; Universidade Federal de Vicosa, BRAZIL

## Abstract

Long distance dispersal to locate suitable breeding sites is recognized as a key trait influencing the population dynamics and distribution of bark beetles and other saprophytic insects. While dispersal behavior has been studied for a range of aggressive ‘tree killing’ bark beetles, few have considered the dispersal behaviour of non-aggressive saprophytic bark beetles that utilize kairomones (host volatiles). We present the results of a mark-recapture experiment that examined adult dispersal patterns of the saprophytic bark beetle *Hylurgus ligniperda*. Releases took place in summer and autumn 2014, in a clearcut pine forest in the central North Island, New Zealand. Both flight-experienced and flight-naïve adults were marked and released in the center of a circular trap grid that extended to 960 m with 170 or 200 panel traps baited with a kairomone blend of alpha-pinene and ethanol. Of the 18,464 released *H*. *ligniperda*, 9,209 (49.9%) of the beetles flew, and 96 (1.04%) of the beetles that flew were recaptured. Individuals were recaptured at all distances. The recapture of flight-experienced beetles declined with dispersal distance, and a diffusion model showed heterogeneous dispersal tendencies within the population. Our best model estimated that 46% of flight-experienced beetles disperse > 1 km, and 1.6% > 5 km. Conversely, no declining pattern was shown in the recapture of flight-naïve beetles, suggesting that emerging *H*. *ligniperda* may require a period of flight to initiate chemotropic orientation behavior and subsequent attraction to traps. We discuss the implications of these findings for the management of phytosanitary risks. For instance, combining landscape knowledge of source populations with dispersal processes facilitates estimation of pest pressure at economically sensitive areas such as harvest and timber storage sites. Quantitative dispersal estimates also inform pest risk assessments by predicting spread rates for *H*. *ligniperda* that has proven establishment capabilities in other countries.

## Introduction

Dispersal is the movement of individuals from their parent source to another location where they may subsequently establish and reproduce [1]. It is a key driver in the temporal persistence and spatial structuring of populations and influences population and ecosystem dynamics, species invasion processes and range shifting (including adaptation to climate change and habitat fragmentation) [1–3].

As a group, bark beetles (Curculionidae: Scolytinae) comprise >6,000 species that exploit woody material and associated fungal tissue from a variety of host tree species [4,5]. Most bark beetles are non-aggressive or saprophytic species that feed exclusively on dead trees (e.g. *Hylurgus* sp.). In contrast, opportunistically aggressive, or secondary species, will preferentially colonize recently killed or weakened trees (e.g. *Tomicus* sp.); whilst primary species regularly attack healthy trees and have either developed a capacity to kill live trees by performing mass attacks (e.g. *Dendroctonus ponderosae* Hopkins), or tolerate host defenses and merely injure host trees (e.g. *Dendroctonus micans* (Kugelann)).

Bark beetles have evolved complex strategies to locate and exploit ephemeral and scattered woody resources in forest environments [6,7]. Their specific habitat requirements have led to strong spatial and temporal structuring of their populations [8–10]. Pre-reproductive dispersal of emerging adult bark beetles is often obligatory because the tissues on which bark beetles develop have usually deteriorated via dying, decay and exploitation by the first larval generation. Flights usually occur at certain periods of the year, and on days when climatic conditions are suitable [11]. During dispersal, the physiological drivers of flight change over time (duration varies between species) as individuals balance the benefits of long distance dispersal with an increasing likelihood of mortality or failure to find suitable host material. From an initial phototactic stage dominated by upward and downwind flight behavior, flight becomes progressively chemotactic where bark beetles orientate towards attractive semiochemicals [12]. Long distance dispersal on convective air currents may occasionally occur, where passive transportation (assisted by active flight) disperses individuals over many kilometers on high-level winds [13–17]. Conversely, active chemotactic bark beetle flight is thought to consist of short-distance flights at relatively low altitude where individuals search for suitable host material or pheromone emitting congeners [18,19].

Two common approaches to quantify dispersal are to estimate the change in local beetle density with distance from a known population source, or from the release and subsequent recapture of marked individuals [20,21]. Mark-recapture requires large numbers of individuals for testing, an efficient method for marking them (which can either be marking live beetles directly or through marking of their breeding material), an effective trapping mechanisms for target species (e.g., semiochemical baits), and appropriate numerical tools for data analysis [21]. Inter- and intra-population variation can confound estimates of individual dispersal capabilities, however this can be overcome by identifying subgroups *a priori*, e.g., by life stages (larvae vs. adults) or life history traits (flight-naïve vs. flight-experienced individuals). The main advantage of *a priori* grouping is the ability to test different populations in parallel. For example, Duelli et al. used distinct marking to identify contrasting dispersal patterns for newly emerged and flight-experienced adult spruce bark beetles, *Ips typographus* (L.) [22]. Heterogeneity in dispersal patterns is usually more difficult to evaluate *a posteriori* (i.e. once all individuals have been communally assessed for their dispersal response), as statistical methods must relate dispersal responses with characteristics of dispersing individuals. For example, Cronin et al., related the size of captured beetles, which is reflective of their sex, with dispersal distance [23]. Interestingly, heterogeneous patterns of dispersal that exceed the expected variability amongst individual flight capabilities can also be observed within a single population. Such patterns are usually characterized by a fraction of individuals being active flyers and dispersing over relatively short distances, contrasting with individuals being at least partially transported passively (e.g. by wind) and dispersing over large distances. What determines the mode of transportation of individuals is difficult to delineate *a posteriori*, as it may relate to adaptive physiological and behavioral responses, or, from a variety of random processes that occur during dispersal.

Release-recapture assays that quantify the dispersal of bark beetles have been performed for a range of species that we summarize in Table A in S1 Appendix.

We present results from a mark-recapture study of the golden-haired pine bark beetle, *Hylurgus ligniperda* (Fabricius), in a clearcut pine, *Pinus radiata* D. Don, plantation in the Central North Island, New Zealand. *H*. *ligniperda* is native to Europe, Russia, Asia Minor, the Mediterranean basin, and the nearby Atlantic Ocean islands, and feeds on recently dead *Pinus* spp. [24–26]. Recently, the species has spread widely beyond its native range and is established in Australia, Japan, New Zealand, South Africa, parts of South America, Sri Lanka, and the U.S.A. [27]. In New Zealand, *H*. *ligniperda* is attracted to freshly cut woody materials, including those from managed *P*. *radiata* plantations and, for this reason may be a quarantine concern for wood exports to some countries [28]. We believe (Table A in S1 Appendix) our study is the first attempt to quantify the dispersal characteristics of a non-aggressive saprophytic bark beetle. Contrasting flight-experienced and flight-naïve adults of *H*. *ligniperda*, we first investigated general patterns and sex-related differences in the density (capture)-distance data using generalized linear models (phenomenological approach). We then compared a series of mechanical models to test for heterogeneity of dispersal tendencies within the flight-experienced beetle population. Although more complex analyses have been proposed [29,30,31], we followed Turchin [21] and explicitly contrasted three alternative models without testing a multitude of models. Parameters from the best mechanistic model also allowed us to estimate the more intuitive dispersal (end points)-distance curves. Suggestions to improve the statistical interpretation of mark-recapture dispersal data are provided 1) by testing different approaches to model long-distance dispersal and 2) by providing confidence intervals over the predicted density-distance and dispersal-distance curves. At last, we discuss how an understanding the dispersal potential of *H*. *ligniperda* can facilitate landscape-level modeling of abundance as a function of distance from known source population (e.g., for estimating the risk of colonization of freshly cut logs in plantation forests).

## Material and methods

### Mark-release-recapture design

**Fig 1:**
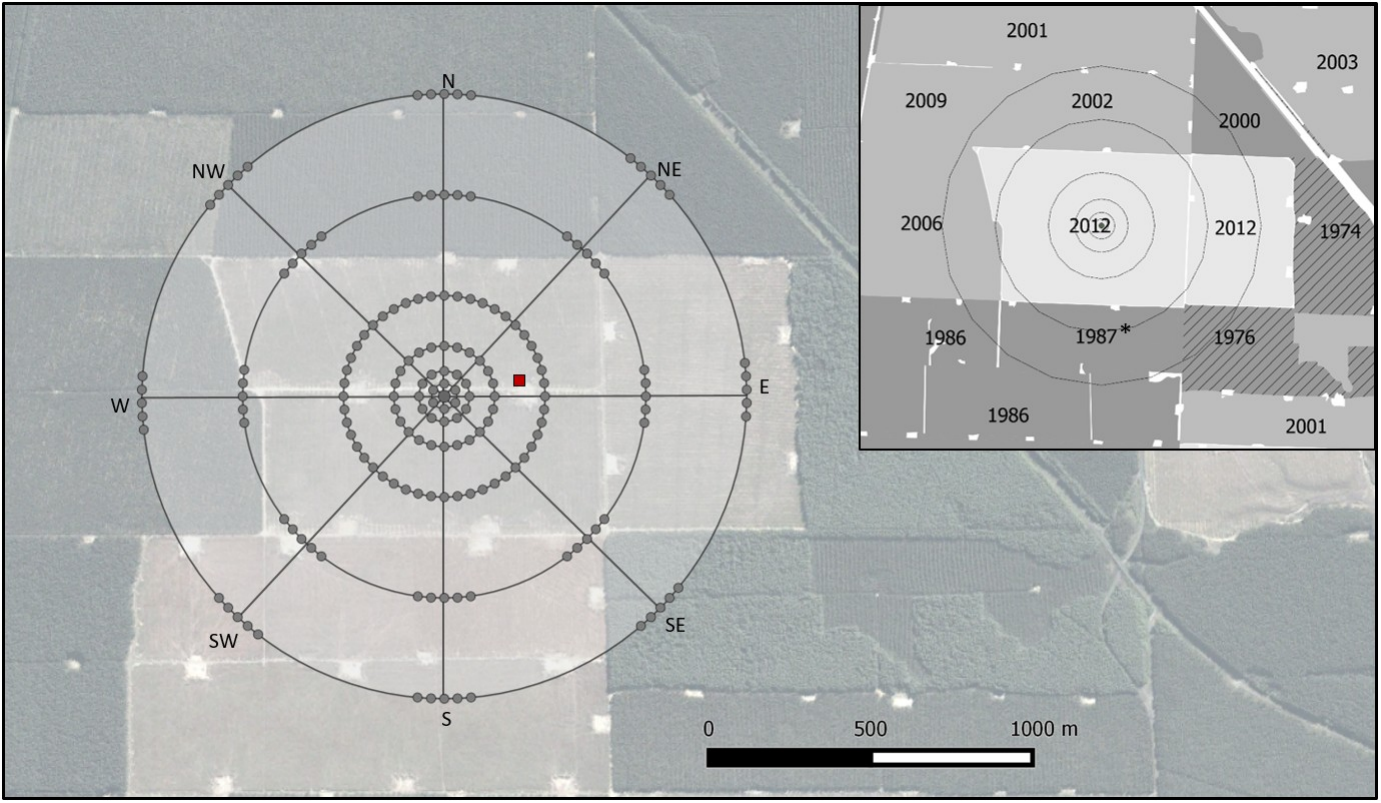
Map of the plantation forest where mark-release-recapture experiments were performed. Dots indicate the position of the traps around the release point (38.4342°S and 176.5178°E). The square symbol indicates the location of the meteorological tower. Inset indicates the year of establishment for each stand. All plantations are *Pinus radiata*, except two *Pseudotsuga menziesii* stands (dashed lines). The stand established in 1987, indicated with a *, had been harvested in March 2013. Source background image. 38.4342°S and 176.5178°E. Google Earth. 23 February 2013. Accessed 27 October 2015.

Mark-release-recapture releases (15 total) were conducted between 3 February and 20 May 2014 in a 161 ha *P*. *radiata* plantation forest stand in Kaingaroa Forest, central North Island, New Zealand (38.43°S, 176.52°E). The stand, which had been clearcut 28–32 months prior, was chosen to minimize the potential influence of host volatiles in the study area. Surrounding stands consisted of *P*. *radiata* (<15 years old or 26–28 years old), and *Pseudotsuga menziesii* (Mirb.) Franco (38–40 years old) (Fig 1). Kaingaroa Timberlands Ltd managed stand and authorized access for the completion of our study.

The *H*. *ligniperda* adults used in our study consisted of two physiologically distinct groups: one group was flight-experienced (beetles that had flown before they were released) and the other group was flight-naïve (beetles that had not flown before they were released). Flight-experienced beetles were obtained from recent clearcuts in Kaingaroa Forest between 23 January and 1 April 2014 using four-vane panel traps baited with a kairomone blend of alpha-pinene and ethanol (Fig A in S2 Appendix). To obtain flight-naïve beetles, 40 freshly-cut *P*. *radiata* logs (about 1m long) were placed in an insect-proof room and infested in October 2013 with *≈*50 adult *H*. *ligniperda* on each log. The adults of the parental generation were obtained from clearcuts in Kaingaroa Forest between 24 September and 8 October 2013 using four-vane panel traps baited with a kairomone blend of alpha-pinene and ethanol. Each log produced *≈*500 flight-naïve adults of the filial generation, which were collected in February–April 2014 by stripping bark from logs (*≈*90% of the beetles) or directly from the room window that attracted walking or flying emerging adults (*≈*10%). The room was not controlled for temperature and had a single small window, so that the temperature inside the room followed, with a light buffering effect, the course of the outside temperature variation. The exact proportion of the window-collected beetles that experienced a short flight to the window is unknown, however given our own observations and the small room size these flights are likely to be limited to very short durations. After they were collected, flight-experienced and flight-naïve beetles were held on moist filter paper in 0.5L plastic containers at 6.0°C in total darkness. The duration of storage was variable but restricted to a few days for most individuals, however it exceeded two weeks for some of the beetles used in releases 2, 3, 4 and 11, and for all beetles in releases 6, 10 and 12 (Table 1).

**Table 1:** Summary of replicate releases and recaptures of *Hylurgus ligniperda*, performed in 2014 in a recent *Pinus radiata* clearcut in central North Island, New Zealand. No strong evidence for unbalanced sex-ratios were detected in the released populations (binomial tests: all *P* >0.01, a Benjamini-Hochberg procedure applied with a false discovery rate of 0.2 indicates none of the tests are significant).

Releases	Number of traps	Flight experience	Time stored (days)	Release date	Sex ratio (M:F)	No. flying	% released that flew	No. recaptured	% flying that were recaptured	Sex ratio of recaptures (M:F)
**1**	170	yes	3–11	03 Feb.	10:17	875	62.1	2	0.2	2:0
**2**	170	no	1–20	03 Feb.	11:8	468	65.1	9	1.9	8:1
**3**	170	yes	4–20	24 Feb.	16:15	1,142	57.2	4	0.4	2:2
**4**	170	no	1–21	24 Feb.	19:12	186	16.1	0	0.0	-
**5**	170	yes	1–7	25 Feb.	- ^b^	1089	55.4	5	0.5	-^d^
**6^a^**	200	yes	18–21	18 Mar.	- ^b^	125	15.7	0	0.0	-
**7**	200	yes	4	18 Mar.	26:24	431	42.9	17^c^	3.9	10:7
**8**	200	yes	9	19 Mar.	25:25	1,110	71.6	34	3.1	19:15
**9**	200	yes	1	19 Mar.	23:29	362	65.6	19	5.2	13:6
**10^a^**	200	yes	30–43	01 May	16:15	90	5.6	0	0.0	-
**11**	200	no	1–48	01 May	21:9	190	9.0	2	1.1	-^d^
**12^a^**	200	yes	>40	02 May	13:17	3	0.4	0	0.0	-
**13**	200	No	1	02 May	18:12	828	37.7	1^c^	0.1	1:0
**14**	200	no	3	19 May	11:26	1,854	85.0	3	0.2	2:1
**15**	200	no	1	20 May	14:22	674	41.8	0	0.0	-

^a^Replicates 6, 10 and 12 were discarded from all analyses, because of low percentage of flying beetles (see Materials and Methods).

^b^Sex-ratios of released beetles are not shown for replicates 5 and 6, as no samples were kept.

^c^91% (87/96) of marked individuals were recaptured within a day after release. However, 7 individuals in release 7 were recaptured on 20 March and 1 individual in release 13 was recaptured on 5 May.

^d^Sex-ratios of recaptures are not shown for replicates 5 and 11, as we were not able to determine the sex of at least one beetle in each of these.

The day before release, insects were bulk-marked by gently shaking them in a polystyrene container containing a fluorescent pigment (Day-Glo, Cleveland, Ohio) at a rate of 0.25 mg per 1,000 beetles. No evidence of an effect of the pigment applied at such rate was observed on the short-term survival and flight capability of *H*. *ligniperda*. Tests showed <5% mortality/day and *≈*80% flight rate for both marked and unmarked beetles. Applications ≥2,000 times higher (0.5 or 1.0 g per 1000 beetles) were required to reduce flight propensity of *H*. *ligniperda* by a noticeable proportion (51% flight rate, 75/148 of marked individuals; 80%, 68/85 of unmarked individuals, χ^2^ = 18.37, *P* = 1.8e-05). The same fluorescent pigments have had no reported short-term effect on flight initiation and semiochemical perception when tested previously on other bark beetle species [19,23,32,33].

Groups of *≈*100 marked *H*. *ligniperda* were placed on moist filter paper in 100-mm-diameter x 15-mm-deep Petri dishes, held overnight at 6 ^°^C, and then kept at 6–16 ^°^C during transport to the release site. Day-Glo’s blue, fire orange, yellow and arc yellow colored pigments were used for successive beetle releases and to distinguish flight-experienced from flight-naïve beetles during the recapture process. The sex ratios of the released populations were estimated on samples of ca. 30 beetles, by inspecting them for the secondary sexual characters on the 7^th^ tergite according to Fabre and Carle [25] and Liu et al. [34] (Table 1).

Marked beetles were released in the center of a grid consisting of alpha-pinene and ethanol baited four-vane black panel traps (Fig 1, Fig A in S2 Appendix) that were placed equidistantly from each other in concentric circles at 40, 80, 160, and 320 m from the center. In the first five releases, all active traps in the four inner circles were separated by ≥40 m by using 6, 12, 24, 48 traps at 40, 80, 160, and 320 m respectively. Low recapture rates were observed in these first five releases and the trapping density was then increased in the two inner rings from 6 to 24 traps, so that the traps in the four inner circles were located 10 m, 21 m, 42 m and 42 m apart respectively for releases 6 to 15. Complete trapping circles at larger distances was not feasible, instead 8 equidistant clusters of 5 traps at 40 m intervals were placed within 640 and 960 m circles (Fig 1). There is a paradoxical situation in mark recapture studies, where one should aim to maximize overall recapture rates to improve statistical analysis but minimize recaptures in traps located close to the release point. This is to prevent depleting of a large proportion of marked individuals by traps near the release point [19,23]. In our study, observed recapture rates remained low despite the increased trap density for releases 6 to 15 (*≈*1% of all flying beetles, see Results).

Meteorological data were collected using a 2.5 m metal tower (Scottech, Hamilton, New Zealand). Data from sensors were recorded on a CR1000 (Campbell Scientific, Logan, USA) data logger with measurements taken every minute. Sensors included RM Young wind monitor, model 05103 (RM Young Company, Michigan, USA); Apogee quantum sun calibration sensor, model sq-110 photosynthetic flux density sensor (Apogee Instruments, Logan, USA); CSI temperature and relative humidity probe, model hc2s3 (Campbell Scientific, Logan, USA); and CSI rain gauge, model tb4 (Campbell Scientific, Logan, USA). The data collected by the sensors are provided in Figs A-E in S3 Appendix.

Marked beetles were released on sunny mornings (air temperature >12.0°C at start of release), with no rain (relative humidity <90%) and limited wind (wind speed <5 m/s). Up to 40% of the beetles were observed to initiate their flight during the coldest releases, when the recorded air temperature (2 m above ground) was between 13 and 15°C. These observations agree with those of Fabre and Carle [25] who observed *H*. *ligniperda* flying at temperature over 15–16°C, corresponding in early spring to 11–13°C at ground level. Pawson et al. observed flight at temperatures as low as 6.3°C with peak flight occurring at 18.6°C [35]. Wind velocity was generally low and only exceeded 2 m/s for releases 10, 11 and 15. Beetles from releases 10 and 11 were characterized by relatively low flight rates (>10%), however a longer period of storage (>10 days for all individuals) prior to that release may also have contributed to the lower flight propensity (see discussion in [26]). Beetles from releases 15 were characterized by moderate flight rate (42%).

**Fig 2:**
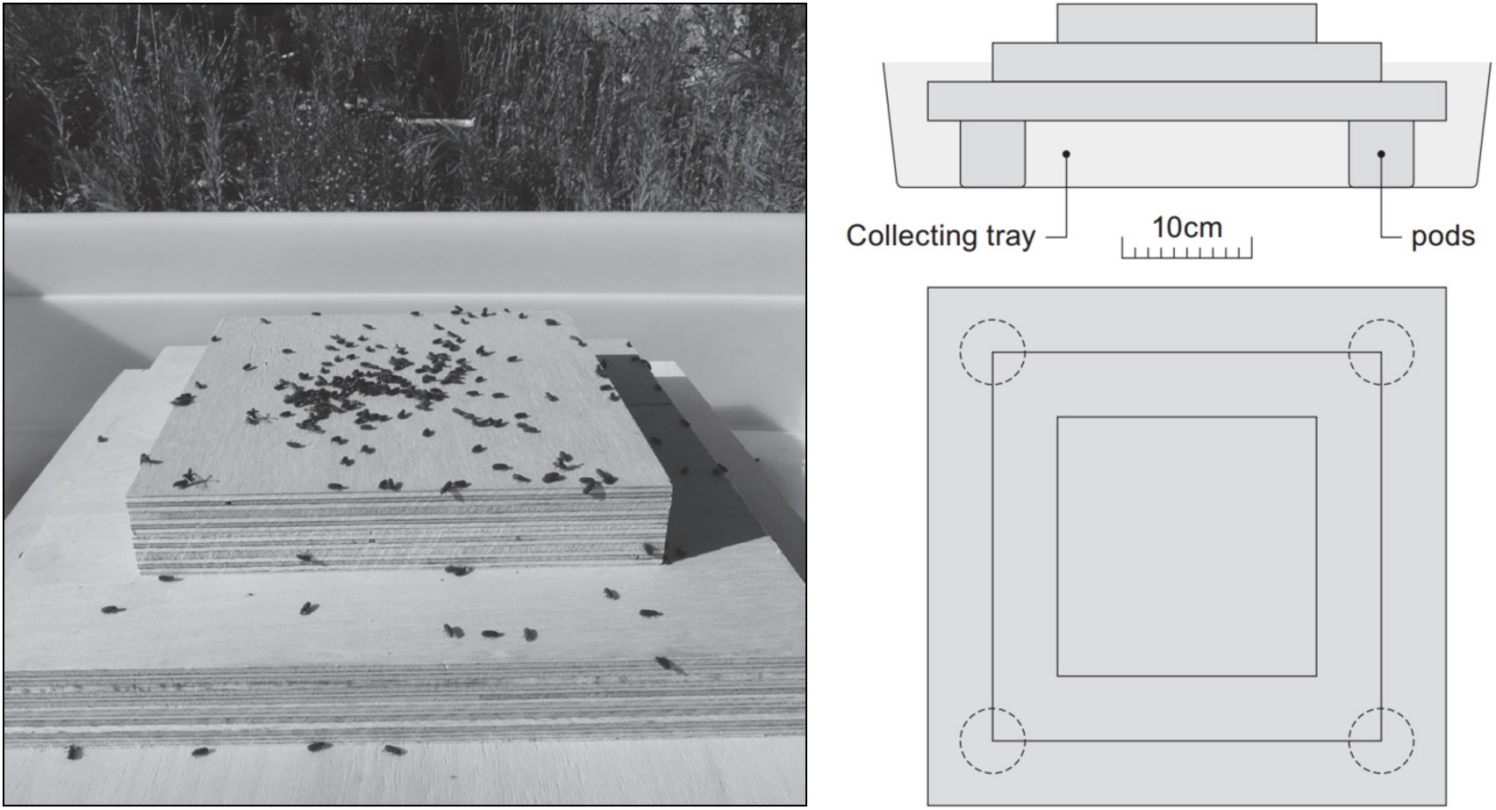
*Hylurgus ligniperda* adults were released from a three-storey pyramidal plywood platform set-up in a collecting tray. The pyramidal shape provided the beetles with a lot of edges that are preferred sites for flight initiation. At the end of each release, immobile dead or moribund beetles were recovered from the platform, while non flying but mobile beetles were recovered in the collecting tray.

The traps were inspected prior to releases to ensure that they were empty and to check for captures from previous releases. In the morning of each release, a known number of beetles were placed on a pyramidal plywood platform (Fig 2) until no additional take-off was noticed for a period of at least 5 minutes (i.e. for about 1h, see Figs A-E in S3 Appendix for detailed timing). At the end of each release the platform was removed and the number of beetles that flew was calculated by subtracting dead or moribund individuals recovered from the platform and the non-flying beetles recovered from the tray beneath the release platform. The proportion of dead and moribund beetles recovered at the end of each release was 1–11% of the beetles initially placed on the platform. Traps were inspected for recaptured beetles the following morning. All captured bark beetles and wood boring insects were counted and determined. Captured *H*. *ligniperda* were examined under an ultraviolet lamp in the laboratory to detect fluorescent pigment on released individuals; they were only considered marked if they had at least two distinct spots marked with dust particles. The sex and size of marked *H*. *ligniperda* were also determined.

### General patterns of dispersal, sex- and size-related differences

Some releases have been delayed due to periods of persistent bad weather, resulting in longer periods of storage (>2 weeks) and pronounced reduction in the proportions of flyers (<20%). We excluded from analysis any releases performed where all beetles had been held >18 day (releases 6, 10 and 12), but retained releases performed with beetles that have been held over a range of short and long durations (releases 2, 3, 4 and 11, Table 1).

The pattern of *H*. *ligniperda* recaptures was examined with generalized linear models (GLM) using a Poisson error structure and a logarithm link function. Models of the log distance from the release point, the sex of recaptured beetles, and their interaction were fitted to the counts of recaptured beetles summed for all traps over each trapping distance (to reduce the effects of stochastic fluctuations and the high proportion of zero trap catches in the original data). To account for varying trap numbers between replicates and across distances, the number of traps was added to the model as a covariable used to denote the sampling intensity (i.e. an offset, [36]). We performed separate analyses for flight-experienced and flight-naïve beetles because their dispersal patterns were highly dissimilar in our tests. Best fit models were selected using Akaike’s information criterion (AIC) [37]. A 95% CI around the prediction was calculated using the quantile of the standard normal distribution as width *W*_*CI*95_ = 2 × 1.96 × *SE*(*P*_*r*_) (calculation performed on the linear predictor then back-transformed). All analyses were performed using the statistical package R, version 3.1.2. for Windows [38].

Size-distance relationships for flight-experienced or flight-naïve beetles was not subject to statistical analysis because of the small number of recaptured beetles. However, a graphical examination based on the maximum pronotum width showed no apparent size, sex, or flight status (experienced and flight-naïve) relationship with distance (Figs A and B in S4 Appendix).

### Directionality

To test the assumption of equal displacement, where beetles disperse equally in all directions, we projected the recapture locations on two transects (north–south and east–west), and tested for each transect whether the net displacement [19] of recaptured beetles across releases was significantly deviating from the release point. We computed weighted mean net displacement and bootstrapped standard errors (se) for each transect, separately for flight-experienced and flight-naïve beetles and assessed the hypotheses that the north–south or east–west mean net displacements were significantly different from zero (release point) using weighted one-sample Student's t-tests [39]. Weights were the number of beetles captured in each release, forced to an average value of 1. Because very few flight-naïve beetles were recaptured, it was not possible to show that the relatively large net displacements observed for that group were significant from zero. We further examined the importance of directional displacements by comparing them to estimates of the spatial scale of dispersal (estimated as the root mean square of the dispersal distances, [21]).

### Diffusion models

Although functional forms used in phenomenological models (e.g., GLM) can be used to describe and predict general patterns, they fail to discriminate detailed components of the dispersal process (e.g., short- and long-distance dispersal) [2,21,40]. We thus fitted dedicated mechanistic models to identify causal mechanisms driving the patterns observed in our GLM phenomenological density-distance model. Mechanistic modeling could only be applied to flight-experienced beetles because they were the only beetles that could be described by a declining dispersal-distance curve.

We used a time-integrative diffusion model [19,21] where the expected number of beetles *N*_*r*_ caught in a trap at distance r from the source can be approximated as Eq 1,

$$N_{r}=Ar^{-1/2}e^{-r/B}$$ (1)

where the negative exponential curve *N*_*r*_ = *ae*^−*br*^ is used for density-distance relationships [21]. This model differs from phenomenological model because 1) the additional multiplier *r*^−1/2^ explicitly considers the dilution effect of diffusion in a two-dimensional space, and 2) the parameter *A* (Eq 2) and *B* (Eq 3) have a biological meaning as combinations of the number of beetles released *N*_*0*_, the effective sampling rate of a trap *α* (m^2^/d), the spatial spread, or diffusion rate, *D* (m^2^/d), and the disappearance rate *δ* (d^-1^) [19].

$$A=\frac{\propto N_{0}}{\sqrt{8\pi }\sqrt[{4}]{D^{3}\delta }}$$ (2)

$$B=\sqrt{D/\delta }$$ (3)

Simple diffusion models (Eq 1) and their derivatives effectively describe insect redistribution processes across a range of species, including bark beetles (see [41], and see S1 Appendix). They are based on the following assumptions: 1) that diffusion with no drift adequately represents the spatial redistribution process, so that beetles move independently of each other and the population redistribution is not influenced by endogenous (e.g., congregation) or exogenous processes (e.g., prevailing winds); 2) the diffusion rate is constant and there is no influence of spatial heterogeneity; 3) the disappearance rate is constant so that individual loss contributions (insects dying, settling down into the substrate or carried away by weather systems) are not explicitly separated; and 4) the effective sampling rate is constant, so that the recapture rate is proportional to the instantaneous and local density of beetles [21]. We estimated *A* and *B* as single constant parameters using nonlinear modeling where A is a scaling parameter proportional to the number of beetles released and the trap efficiency, and *B* is a measure of the spatial scale of dispersal. These two parameters were later used as input parameters to determine the cumulative distribution of dispersal distances (see below). We did not attempt to provide estimates for the three mechanistic parameters comprising *A* and *B* (*α*, *D* or *δ*), as Eqs 2 and 3 only provide us with a system of two equations with three unknowns [21].

To account for the variation in the total number of organisms released between each replicate experiment, we fitted our models using the proportion of recaptured beetles *Pr* rather than the actual number of recaptured beetles *Nr* [23].

We started with a simple diffusion model (Eq 4) where *A*′ is normalized by N_0_ (Eq 5).

$$P_{r}=A\prime r^{-1/2}e^{-r/B}$$ (4)

$$A^{\prime }=\frac{A}{N_{0}}=\frac{\propto }{\sqrt{8\pi }\sqrt[{4}]{D^{3}\delta }}$$ (5)

To account for stronger leptokurtosis and possible variation in dispersal among individuals [42,43] we used a mixture model that accounts for both short-distance (SD) and long-distance (LD) population dispersal [40,44]. The mixture model considers heterogeneous dispersal that could result from within population differences in terms of dispersal capability, dispersal mechanisms or settling responses [23,30]. It applies as the sum of two diffusion models, accounting for two subgroups of beetles with different dispersal capabilities [23,45], Eq 6, here after referred to as the heterogeneous diffusion model.

$$P_{r}=A_{1}\prime r^{-1/2}e^{-r/B_{1}}+A_{2}\prime r^{-1/2}e^{-r/B_{2}}$$ (6)

The parameters *A*_1_′ and B_1,_ and *A*_2_′ and B_2_, respectively account for SD and LD dispersers.

Following the work of Turchin [21], we evaluated another mixture model with a flat tail LD dispersal component as a null model (Eq 7, referred to hereafter as the mixed diffusion and equal redistribution model).

$$P_{r}=A\prime r^{-1/2}e^{-r/B}+C\prime$$ (7)

The parameter *C*′ corresponds to the proportion of the released beetles whose recapture probability is independent of the distance to the emergence site. *C*′ is equal to *C/N*_*0*_, where *C* is proportional to the trap effective sampling rate α and the local density of the LD dispersing beetles *ρ* (Eq 8). *C* in Eq 8 is also analogous to the number of beetles captured in a single trap [46].

$$C^{\prime }=\frac{C}{N_{0}}=\frac{\propto \rho }{N_{0}}$$ (8)

We emphasize this model may not reflect biological reality if applied to predictions over large areas, as the function does not tend to zero within the range of the maximum dispersal distance. However, at the landscape level we can conceptualize this model as a subgroup of LD dispersing beetles that spread out of the release area and mix homogeneously in the landscape before they are attracted to traps. Therefore, we introduced the concept of the local density of the LD dispersing beetles ρ (numbers per unit area), which can be considered as the number of beetles in the subpopulation that disperse long distances divided by the area that they occupy.

Model parameters were estimated by fitting nonlinear models using generalized least squares [47]. Heteroscedasticity was detected in model validation plots of the residuals vs. the fitted values and accounted for by an exponential or power variance that compensated for the decreasing variance with increasing absolute fitted values [48]. We used the AIC to compare model performance taking both descriptive accuracy and parsimony into account [37], and likelihood ratio tests to determine if a model was significantly better performing than another one [36]. Akaike weights were computed from raw AIC values, which can be directly interpreted as the weights of evidence that an individual model is actually the best model given the data and the set of candidate models [49]. Recapture predictions and symmetrical 95% confidence intervals (CI) with width $W_{CI95}=2\times 1.96\times \sqrt{Var(P_{r})}$ were computed around the predictions from the best model using variance calculation based on Taylor series expansion [50]. A 95% confidence interval around the prediction was calculated on the linear predictor using the quantile of the standard normal distribution as width *W*_*CI*95_ = 2 × 1.96 × *SE*(*P*_*r*_), then back-transformed.

### Distribution of dispersal distances

The dispersal kernel (cumulative distribution of dispersal distances end points) provides a more intuitive description of dispersal that indicates the proportion of beetles (x) dispersing from a source and settling at any given distance up to *r*_*max*_ [21,51]. Using the dispersal quantiles method [19,23], we iterated Eq 9 for the best model (heterogeneous diffusion model, Eq 9) across all *r*_*max*_ values ranging from 40 to 10,000 m to provide an overview of the dispersal process for *H*. *ligniperda* at the landscape level.

$$x_{\left(r_{max}\right)}=\frac{A_{1}\prime \int_{0}^{r_{max}}r^{1/2}e^{-r/B_{1}}+A_{2}\prime \int_{0}^{r_{max}}r^{1/2}e^{-r/B_{2}}}{A_{1}\prime \int_{0}^{\infty }r^{1/2}e^{-r/B_{1}}+A_{2}\prime \int_{0}^{\infty }r^{1/2}e^{-r/B_{2}}}$$ (9)

Confidence intervals (95% CI) for all estimates of x were obtained using the jackknife procedure, by dropping one individual observation from the dataset at a time [52]. We estimated the corresponding four parameters *A*_*1*_, *B*_*1*_, *A*_*2*_ and *B*_*2*_ by fitting nonlinear models over each jackknife sample, then iterated Eq 9 for each value of *r*_*max*_ over each set of the jackknifed estimated parameters. The range of values enclosing the 0.025 and 0.0975 quantiles for x represented the 95% CI.

## Results

### General patterns of dispersal and sex-related differences

There was strong variation between replicates of the percentage of *H*. *ligniperda* that flew and were recaptured (Table 1). Of the 18,464 beetles released across all retained replicate releases, 49.9% flew, of which, 1.04% (96 individuals) were recaptured in our trapping grid. This provides relatively few captured individuals for statistical analysis, but it is also desirable as it overcomes two common drawbacks in single-release-multiple-trap recapture experiments. First, the low recapture rates indicate that that there was no significant depletion of dispersing beetles by the traps closest to the release point that would bias the probability of recapture at larger distances [19,23]. Second, no reduction in the average catch per trap was observed with increasing trap density. The overall average catch per trap was similar in the 170 traps and 200 traps designs (0.006% and 0.009% of the released population respectively), while the proportion recaptured at 40 m was even slightly higher in the highest trap density configuration (0.02% recapture with 6 traps, 0.04% with 24 traps). This suggests closely-spaced traps do not interfere or only do so by a small amount, hence the recapture probability of a given trap does not have to be adjusted for overlapping regions of attraction [53].

Males and females were recaptured at all distances, including the outer ring of 40 traps located 960 m away from the release point. At this maximal distance, a single trap captured one male from the flight-experienced population (0.02% recapture from 5,009 individuals flown) and another trap captured one female from the flight-naïve population (0.024% recapture from 4,200 individuals flown).

**Fig 3:**
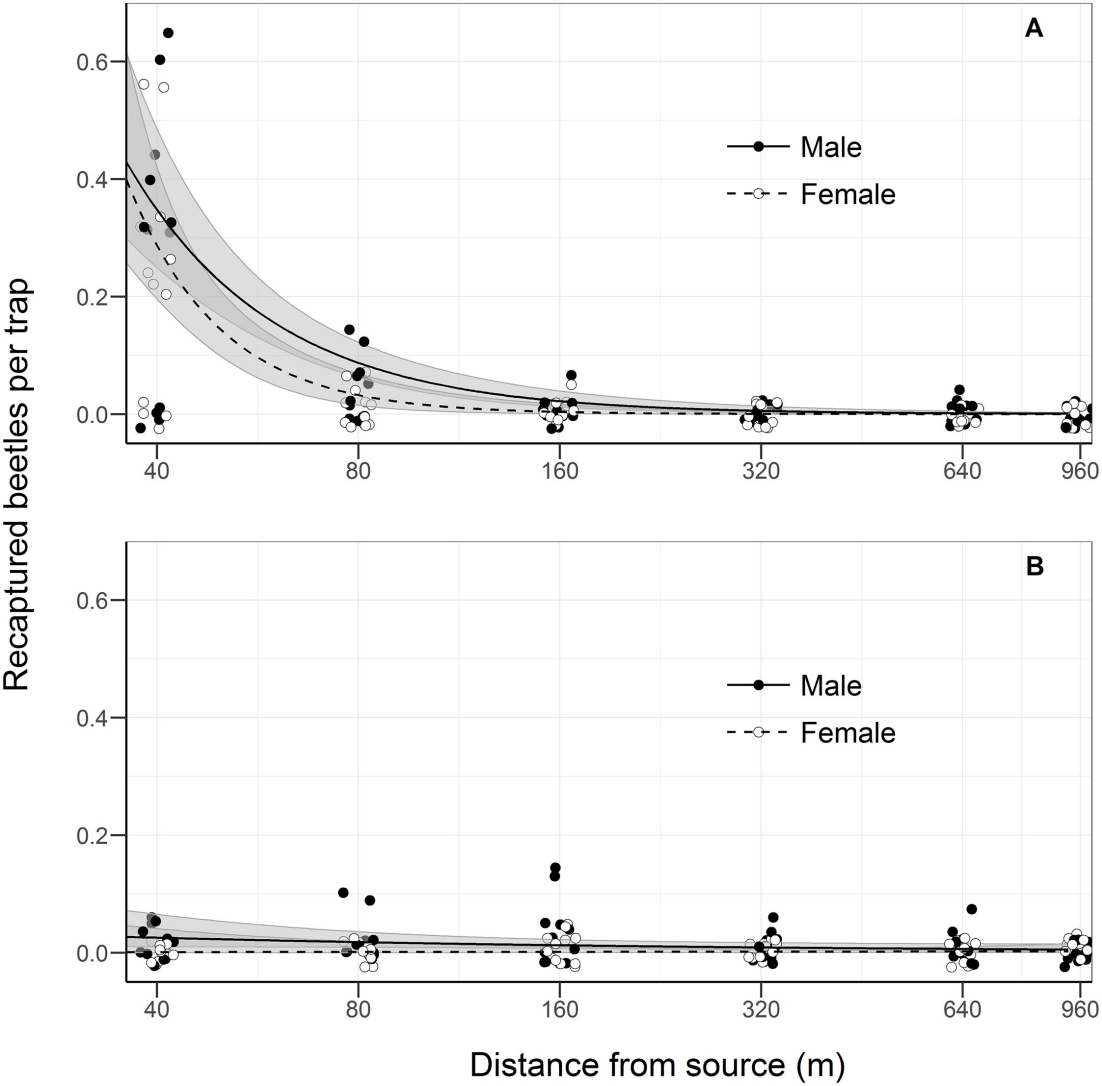
***Hylurgus ligniperda* recaptures with distance for (A) flight-experienced beetles and (B) flight-naïve beetles.** There were six experimental releases in each flight experience category. Curves (lines) and 95% CI (shaded areas) are based on the generalized linear models (GLMs) with a Poisson error structure and a logarithm link function. Only the full models are shown, i.e. the models relating the counts of recaptured beetles summed over each trapping distance to the logarithm of the distance from the release point, the sex of recaptured beetles, and their interaction. Parameter values are provided in Table 2. Note that the x-axis is on a log 10 scale.

A clear pattern of declining recapture with distance dispersed was observed for flight-experienced beetles (Fig 3) with the corresponding percentage recapture of flying beetles averaging 0.067% (40 m), 0.011% (80 m), 0.0025% (160 m), 0.00083% (320 m), 0.00050% (640 m) and 0.00050% (960 m) per trap. On the other hand, no clear pattern of declining recapture was observed for flight-naïve beetles, characterized by a fairly constant recapture rate across all distances around 0.002% per trap.

Phenomenological modeling confirms this via a highly significant distance effect, and a marginal sex-related difference in the dispersal distance for flight-experienced beetles (Table 2). Conversely, distance only marginally influenced the recapture rate of flight-naïve beetles (GLM, Poisson, *Χ*_1_ = -2.72, *P* = 0.099), however a male recapture bias was observed (GLM, Poisson, *Χ*_1_ = -7.92, *P* = 0.0049, overall sex ratio was 12♂:2♀, 86%, binomial test, *P* = 0.013).

**Table 2:** Results of a generalized linear model of recaptured *Hylurgus ligniperda* as a function of distance to release site and sex.

Model	AIC	Variables included	Estimate	Std. error	z value	Pr(>|z|)	df
**Flight-experienced beetles**							
**Full model**	123.6	Intercept	10.3	2.22	4.65	3.3e-6***	-
		log(Distance)	-3.14	0.58	-5.39	6.9e-8***	1
		Sex	-4.04	2.44	-1.66	0.0975^.^	1
		log(Distance) x Sex	1.15	0.63	1.81	0.0695^.^	1
**Distance + Sex**	125.8	Intercept	7.05	0.93	7.62	2.5e-14***	-
		log(Distance)	-2.23	0.23	-9.95	<2e-16***	1
		Sex	-0.43	0.23	1.82	0.0685^.^	1
**Distance only**	127.2	Intercept	7.29	0.91	8.0	1.6e-15***	-
		log(Distance)	-2.29	0.23	-9.95	<2e-16***	-
**Flight-naïve beetles**							
**Full model**	79.8	Intercept	-7.96	4.70	-1.69	0.0904^.^	-
		log(Distance)	0.28	0.78	0.36	0.72	1
		Sex	6.18	4.90	1.26	0.21	1
		log(Distance) x Sex	-0.79	0.82	-0.96	0.34	1
**Distance + Sex**	78.9	Intercept	-4.10	1.47	-2.79	0.00521**	-
		log(Distance)	-0.41	0.24	-1.67	0.0942^.^	1
		Sex	1.79	0.76	-2.35	0.0190*	1
**Sex only**	79.6	Intercept	-6.35	0.71	-8.97	<2e-16***	-
		Sex	1.79	0.76	-2.35	0.019*	1

Significance is indicated as P < 0.10

* P < 0.05

** P < 0.01, and

*** P < 0.001. No strong evidence for bias was detected in the overall sex-ratio of flight-experienced beetles recaptured (46♂:30♀, 61%, binomial test, *P* = 0.08).

### Directionality

**Fig 4:**
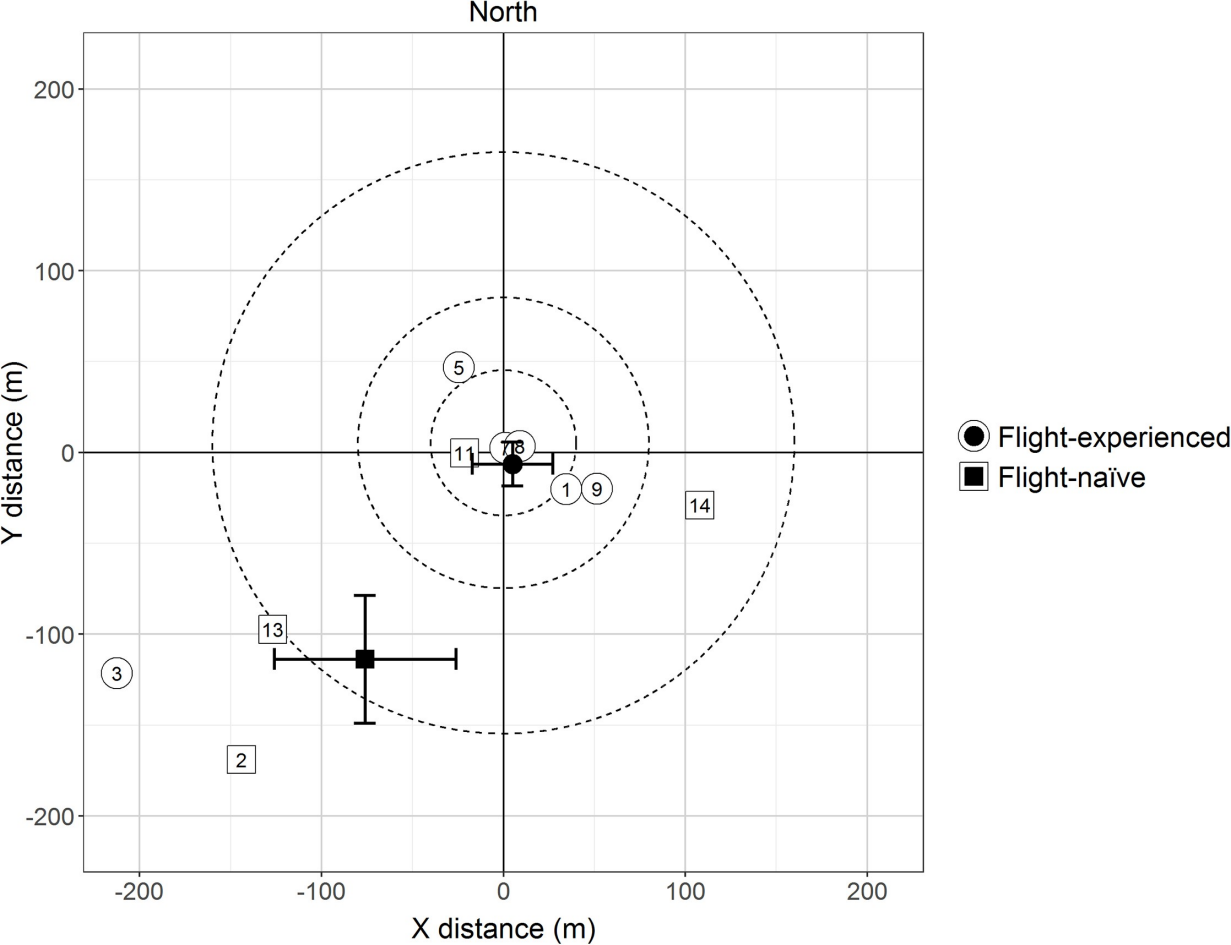
Net displacement of released *Hylurgus ligniperda*. The open circles (flight-experienced) and squares (flight naïve beetles) represent the mean net displacements for each release. The closed circles (flight-experienced) and squares (flight naïve beetles) represent the mean net displacement for pooled releases (±1 se).

Wind speed and direction varied between and within an individual release day (Figs A and B in S3 Appendix). Marked individuals were captured in the inner trap rings in all directions, while none of the 5 individuals in the outer trap clusters were captured in the northern part of the study area (Figs A-C in S5 Appendix). Mean (±se; t-test) net displacements were 5 m eastwards (± 23 m; *t* = 0.22, df = 5, *P* = 0.84) and 6 m southwards (± 13 m; *t* = -0.50, df = 5, *P* = 0.64) for the flight-experienced beetles, and 76 m westwards (± 50 m; *t* = -1.51, df = 3, *P* = 0.23) and 114 m southwards (± 34 m; *t* = -3.33, df = 3, *P* = 0.045) for the flight-naïve beetles (Fig 4), indicating that southward directionality, or drift, was only marginally significant for the flight-naïve beetles. Comparisons with the estimated spatial scales of dispersal suggested no drift for the flight-experienced beetles (3% to the east and 4% to the south, over a dispersal scale of 148 m), and a significant southwestern drift for the flight-naïve beetles (20% to the west and 30% to the south, over a dispersal scale of 376 m).

In addition to wild individuals of *H*. *ligniperda*, another bark beetle, *Hylastes ater* (Paykull) (Coleoptera: Scolytinae), and two wood boring species, *Arhopalus ferus* (Mulsant) (Coleoptera: Cerambycidae), and *Sirex noctilio* Fabricius (Hymenoptera: Siricidae), have also been regularly captured in the traps. The spatial distribution of their captures (Figs D-F in S5 Appendix) indicates most individuals were caught in the center and in the southern part of the study area. The central stand in the study area is unlikely to be the source for these beetles, as it had been harvested 2½ year before releases and was thus containing already exploited or no further suitable woody debris. A stand that had been harvested 1 year before releases and nearby mature stands experiencing natural tree mortality (pers. obs.), all located in the southern part of the study area, were the most likely source for the captured insects. *H*. *ater* has also been captured in abundance in the northernmost traps cluster located in a young plantation, where woody debris has been produced by recent pruning and thinning operations.

### Diffusion models and distribution of dispersal distances

We could only apply diffusion models to flight-experienced beetles as they exhibited the necessary observed difference in recapture rate as a function of distance.

**Fig 5:**
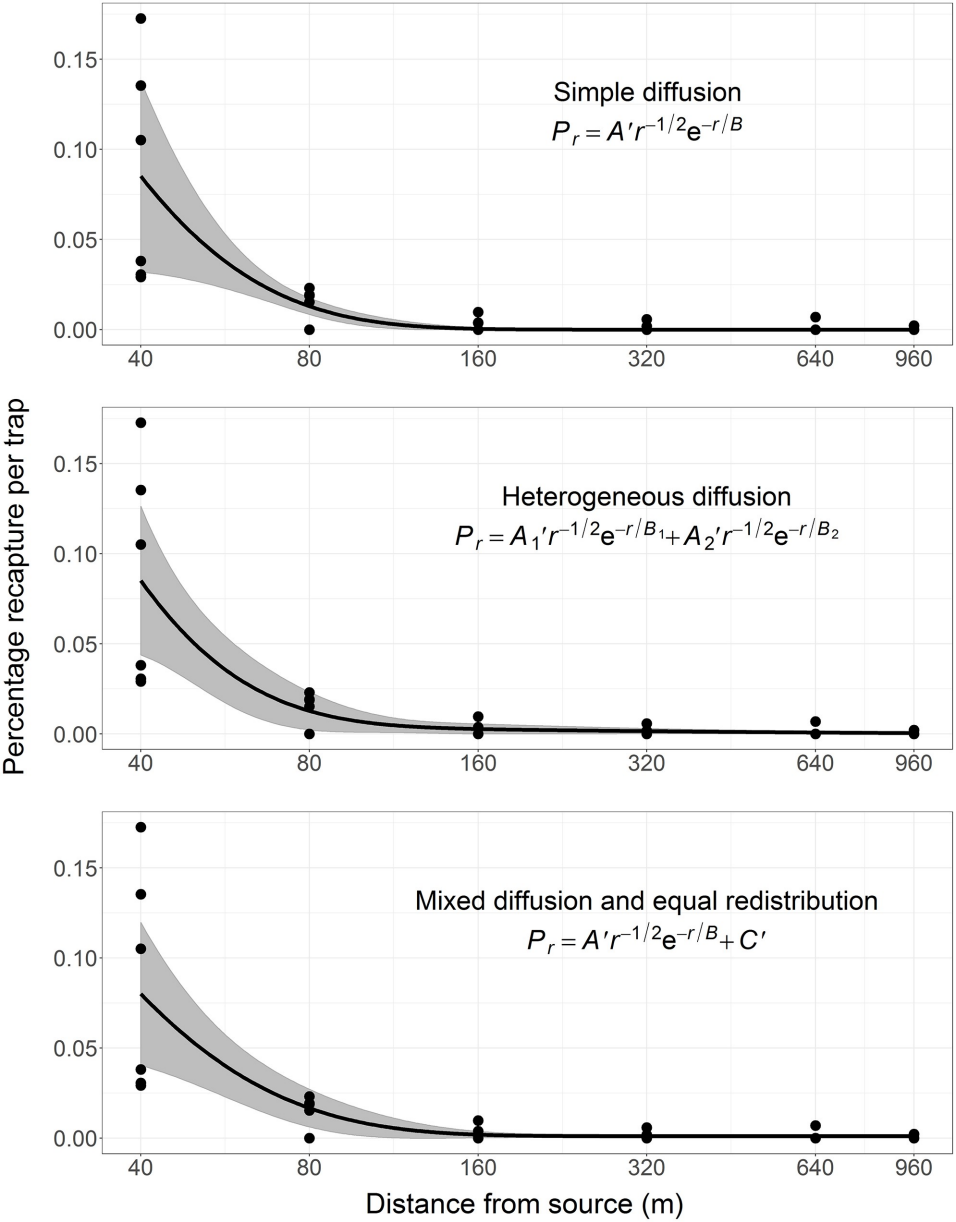
*Hylurgus ligniperda* recaptures with distance for the six experimental releases (flight-experienced beetles). Recapture rates (dots), distance curves (lines) and 95% CI (shaded areas) for the simple diffusion model with an exponential variance function, the heterogeneous diffusion model with a power variance function, and the mixed diffusion and equal redistribution model with a power variance function. Parameter values are provided in Table 3. Note that the x-axis is on a log 10 scale.

The dispersal-distance curve for flight-experienced *H*. *ligniperda* (Fig 5) was characterized by a leptokurtic distribution (i.e. a “fat” tail) that indicates differences (heterogeneity) in the dispersal capability, dispersal mechanisms and/or settling response of individuals. All diffusion models were improved by exponential or power variance modeling, and the heterogeneous diffusion model with a power variance was the best model (Table 3). The heterogeneous diffusion model was superior to: (1) the simple diffusion model which indicates there is probably more than one dispersal mechanism; (2) the mixed diffusion and equal redistribution model (i.e. a null model for the LD dispersing population) which indicates the tail is not completely flat but decreases with distance. Akaike weights indicate that the heterogeneous diffusion model with a power variance function is 3.8 times more likely to be the best model than is the next-best model, the mixed diffusion and equal redistribution model (weights ratio = 0.79 / 0.21).

**Table 3:** Results of diffusion models on the recapture rates of *Hylurgus ligniperda* depending on distance to release site (flight-experienced beetles).

Model and variance function	AIC	Akaike weight	Parameter	Estimate	CI 0.025	CI 0.975
**Simple diffusion**						
**None**	-162	6.2e-24	*A*′	2.5	-1.5	6.5
			*B*′	26.1	-0.2	52.3
**Exponential**	-255	7.2e-4	*A*′	2.5	-1.0	5.8
			*B*′	26.1	13.6	38.6
**Power**	-237	8.01e-8	*A*′	2.5	-2.0	7.0
			*B*′	26.1	-3.0	55.1
**Heterogeneous diffusion**						
**None**	-158	8.8e-25	*A*_1_′	3.2	-9.0	15.4
			*B*_1_	21.6	-30.0	73.0
			*A*_2_′	0.04	-0.43	-0.51
			*B*_2_	1047	-41,689	43,784
**Exponential**	-254	5.4e-4	*A*_1_′	3.2	-2.2	8.6
			*B*_1_	21.6	8.7	34.4
			*A*_2_′	0.04	0.03	0.10
			*B*_2_	1047	-5121	7215
**Power**	-269	0.791	*A*_1_′	3.2	-2.4	8.7
			*B*_1_	21.6	4.02	39.2
			*A*_2_′	0.04	0.02	0.10
			*B*_2_	1022	-2130	4174
**Mixed diffusion and equal redistribution**						
**None**	-160	2.4e-24	*A*′	2.7	-2.6	8.0
			*B*′	24.4	-4.7	53.6
			*C*′	0.001	-0.009	0.012
**Exponential**	-253	3.5e-4	*A*′	2.7	-1.2	6.6
			*B*′	24.4	12.4	36.4
			*C*′	0.0005	-0.001	0.002
**Power**	-266	0.207	*A*′	1.8	-2.7	3.9
			*B*′	31.3	11.7	50.9
			*C*′	0.001	0.00005	0.002

**Fig 6:**
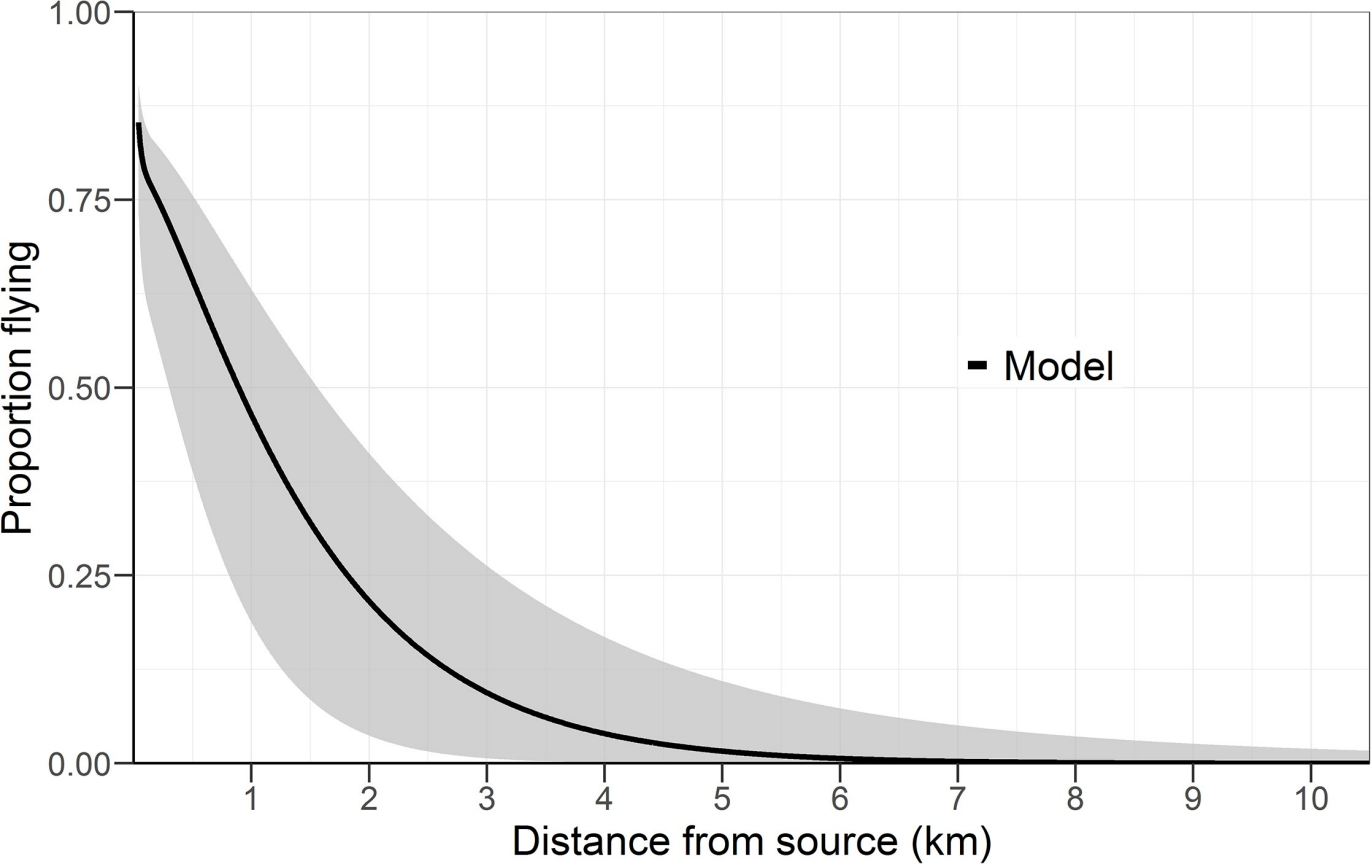
*Hylurgus ligniperda* distribution of dispersal distances. Distribution curve (line) and 95% CI (shaded area), as estimated with Eq 9 from the parameters A_1_′, B_1_, A_2_′ and B_2_ from the heterogeneous diffusion model with a power variance function (Eq 6, Table 3).

Applying the parameters A and B from the heterogeneous diffusion model show that 77% of released beetles disperse > 100 m on average, whereas 46% disperse > 1 km (CI_95_: 19%–63%), and 1.6% over 5 km (CI_95_: 0.02%–11%) (Fig 6).

## Discussion

### Recapture rates and dispersal range

At short distances, recapture rates of marked *H*. *ligniperda* were higher for flight-experienced (0.067% per trap at 40 m) than flight-naïve beetles (0.002%). Variable but overall higher recapture rates have been observed at similar short distances for other scolytines. In two separate experiments using traps baited with alpha-pinene, Poland et al. [54] recaptured emerging individuals of the moderately aggressive *Tomicus piniperda* (L.) in relatively low proportions (0.015% and 0.026% per trap at 50m), comparable to our observations with *H*. *ligniperda*. The other studies we have reviewed were assessing the dispersal of more aggressive bark beetles species for which species-specific pheromone lures (S1 Appendix) resulted in slightly higher recapture rates. Short distance recapture rates for flight-experienced beetles ranged between 0.16% (*Trypodendron lineatum* (Olivier) in the 30–200 m range, [55]) and 6.2% per trap (*Dendroctonus pseudotsugae* Hopkins at 50 m, [56]), and for flight-naïve beetles between 0.022% (*Dendroctonus frontalis* Zimmermann at 50 m, [19]) and 6.2% per trap (*D*. *pseudotsugae* at 50 m, [56]).

Greater recapture rates of aggressive species (*sensu* Weed et al. [57]) reflect the high catching power of pheromones, which typically serve aggregation processes where congeners are drawn to a specific host trees by others that have already commenced burrowing [12,58]. Lower recapture rates compared to aggressive species are expected for *H*. *ligniperda* as it relies on short-distance perception of kairomones/apneumones emitted from dying, injured or fallen trees and the physical/visual stimuli provided by the trap [59].

The higher catching power of pheromone traps for aggressive species is also well evidenced at long distances. The minute fraction of released *H*. *ligiperda* recaptured at the furthest trapping distance of 960 m (0.0005% per trap for flight-experienced, and 0.0006% per trap for flight-naïve beetles) was about one to three orders of magnitude less than rates observed at similar distances for aggressive bark beetle species, ranging from 0.007% (flight-naïve *Scolytus multistriatus* (Marsham) at 1,500 m, [60]) and 0.009% (flight-naïve *D*. *frontalis* at 1,000 m, [19]), to 0.19% (flight-experienced *Ips typographus* at 1,000 m, [61]), and 0.49% (flight-naïve *Ips sexdentatus* (Boerner) at 1,000 m, [62]). These observations have been all made using traps baited with pheromones or a mixture of pheromones and kairomones. Unfortunately few studies report mark-recapture of bark beetles performed using traps baited with non-pheromone semiochemicals as a basis for comparison. Poland et al. [54], in their attempts to recapture *T*. *piniperda* with alpha-pinene baited traps, did not capture any of the 2,117 adults released at the maximal distance tested of 400 m, and only managed to capture some in traps ≤ 250 m as well as in uninfested pine logs ≤ 100 m.

We captured flight-experienced and flight-naïve *H*. *ligniperda* up to a distance of 960 m from their release point, and our best model estimated that 46% of flight-experienced beetles disperse over 1 km, and 1.6% over 5 km. These are consistent with observations by Fabre and Carle [25] who described *H*. *ligniperda* adults to be capable of flight over several kilometers.

### Required flight period for chemotactic response

Flight-naïve *H*. *ligniperda* were characterized by low recapture rates at all distances, with only a small peak observed in the 160 m ring. This suggests that flight-naïve *H*. *ligniperda* require a period of flight before chemotropic orientation occurs. This is consistent with the transition from phototactic to chemotropic behaviour documented in other bark beetles after a certain flight duration, e.g. *Dendroctonus pseudostsugae* [63,64]; *Ips sexdentatus* [62]; *Ips typographus* [22,65–68]; *Scolytus multistriatus* (Marsham), [69]; *Tomicus piniperda* [70]; *Trypodendron lineatum* [64]. Poland et al. [54], also observed a similar, relatively flat capture-distance pattern in rings of trap set up around a source of emerging *Tomicus piniperda*. Interestingly, the same experiment testing the colonization of freshly cut pine logs set up at increasing distances did well exhibit the declining with distance curve [54] typically observed in mark-recapture studies [41].

Studies directly comparing captures of flight-experienced and naïve individuals are relatively scarce in scolytines. Wollerman [71] simultaneously released *S*. *multistriatus* that were previously exposed to a variety of food and flight regimes, and observed higher response to pheromones in 24hr-old, flight-experienced, individuals (at 32 m, 0.083% recapture per trap) compared to flight-naïve individuals (0.025%). Before synthetic pheromone lures were available, Gara [72] used traps baited with infested pine logs to capture marked *Ips paraconfusus* Say. Comparing flight-experienced and flight-naïve individuals, he observed similar recapture rates for both groups at short distance (e.g. 15.2% and 14.2% per trap at 5 m, respectively) and at long distance (e.g. 0.26% and 0.14% per trap at 1,000 m, respectively), but flight-experienced beetles were more sensitive than flight-naïve ones at intermediate distance (e.g. 3.3% and 1.2% per trap at 50 m, respectively). Most other studies were generally not performed at the same time and location, or over an extended range of distances (e.g. [22,56,73]), and to our knowledge, there are no published studies investigating the effect of previous flight experience on dispersal of saprophytic bark beetles.

### Heterogeneous dispersal in flight-experienced individuals

The reasons for the observed heterogeneity in the dispersal of flight-experienced *H*. *ligniperda* still need to be elucidated. We examine here the most likely, non-exclusive, explanations.

First, heterogeneity in the developmental and post-emergence life history of beetles, or diverging adaptive responses, may condition particular life traits affecting dispersal. These could affect individual bark beetle flight capability (energetic resources, wing muscles development), and response to host volatiles (reproductive system development, response to light, and other visual or chemical stimuli) [12,13,22,23,62]. We showed in our study the importance of previous flight experience on the dispersal pattern of *H*. *ligniperda*, whereas only marginal (flight-experienced beetles) or low significance (flight-naïve beetles) sex-related differences were detected. We believe it is unlikely that physiological or morphological differences are large enough to explain the strong leptokurtosis character observed in the dispersal-distance curve.

The physical and chemical landscape influences bark beetle dispersal, as it may affect the disappearance rate of beetles, and individual trap efficiency [21,41,74]. In mark-recapture experiments, the need to capture a sufficiently large number of beetles for statistical analysis has to be balanced with the need to minimise competition between individual traps (especially in the vicinity of the release point). Additionally, too many traps can breakdown natural dispersal processes with beetle movements directed solely by the response to odor sources on traps. Therefore, to adequately represent dispersal processes and beetle behavior in the wider environment, a mark-recapture study should be designed with either a restricted number of traps with high catching power (e.g. pheromone traps, in particular in the vicinity of the release point), or a large number of traps with low catching power (e.g. kairomone traps).

Our releases were centered on a large plantation stand clearcut 28–32 months prior, this provided a relatively homogeneous physical and chemical landscape to the dispersing beetles. Large amounts of coarse woody debris were still present that could have affected dispersal by providing visual and physical cues to the flying beetles. However, these were unlikely to emit large amounts of host volatiles that could have competed with the lure chemical blends. The absence of directionality in dispersal of the flight-experienced *H*. *ligniperda* still suggests that the effect of environmental heterogeneity are likely to be minor in our study.

A third explanation is that a portion of the dispersing beetles may experience another dispersal mechanism in addition to active flight. For instance, a small fraction of the population may exhibit a strong phototropic response and occasionally become transported by wind over long distances, before they begin to exhibit chemotactic responses [12,15,16,75]. This strategy, which is seen to be successful for species that exploit spatially ephemeral resources, such as stumps, roots and other parts of recently dead wood, has been previously documented and referred to as a “spread out, then search” strategy [9]. It is experimentally characterized by steeper catch by elevation profiles [12,76]. Nevertheless, the development of both phenomenological and mechanistic models that accurately reflecting long distance dispersal events remains an issue, in part, due to the limited spatial scale of mark-release-recapture sampling designs [51]. Hence we cannot estimate the strength of the ‘spread out and search’ strategy for *H*. *ligniperda*. In some anecdotal instances, individual bark beetles and weevils are reported to have been wind-transported over particularly long distances. For instance, *Dryocoetes autographus* (Ratzenburg), *Hylastes cunicularius* Erichson, *Hylastes brunneus* Erichson (Coleoptera: Scolytinae) and *Hylobius abietis* (L.) (Coleoptera: Curculionidae) have been recorded as far as 171 km from the closest forest source [75,76]. In New Zealand, both *Hylurgus ligniperda* and *Hylastes ater* (Paykull) have been trapped ≥25 km from the nearest host patch. Still, such records are characterized by a high degree of stochasticity, and no clear evidence regarding the dispersal mechanisms involved (active flight vs. wind-aided or human-aided dispersal by the movement of firewood and timber) [77], with distance probably being a minor and inaccurate predictor of the dispersal probability of individuals.

### Other factors influencing dispersal in *H*. *ligniperda*

Our experiments, like most mark-recapture experiments, were performed under particular conditions (one particular forest, summer season) and the generality of our findings to different populations, regions, or seasons is unknown. For example, populations of *Dendroctonus frontalis* disperse further during spring and fall months, compared to summer [19]. The number of *H*. *ligniperda* generations that occur each year is variable and there is a corresponding emergence of young adults and re-emergence of adults after the initial egg-laying from late winter or early spring to autumn [26,78,79]. Usually two main peaks of flight activity are observed in New Zealand with one flight in spring and another in autumn. Whether the beetles in the two peaks of flight activity have different dispersal patterns is unknown. In addition to potential weather and/or seasonal influences, our dispersal capability calculations do not consider the potential for fragmented dispersal occurring due to flight on successive days. Our estimates were based on individuals that had already flown, thus we only monitor their most recent dispersal activity, and cannot calculate the total distance they travelled. Most recaptured individuals were potentially capable of further flight after trapping. Thus our estimates of *H*. *ligniperda* dispersal will be most accurate for beetles that settle once they have landed after chemotropic response, however they may slightly underestimate the dispersal of individuals that undertake multiple flights.

### Recommendations for management

Estimated probabilities of dispersal for flight-experienced *H*. *ligniperda* show 50% of them may disperse over 0.89 km, 33.3% over 1.45 km, 5% over 3.73 km, and 1% over 5.55 km. These estimates are comparable to estimates made using similar methods for flight-naïve *D*. *frontalis* (50% over 0.69 km, 33.3% over 0.99 km, 5% over 2.27 km, 1% over 3.29 km, [19]) and flight-naïve *Ips grandicollis* (Eichoff) (50% over 1.54 km, 33.3% over 2.29 km, 5% over 5.42 km, [45]). Duelli et al. [22] estimated lower rates of long-distance for *Ips typographus* with 1% of the flight-naïve and flight-experienced individuals dispersing over 1.56 km and 0.34 km, respectively.

Knowledge of the dispersal potential of a species is a prerequisite to modelling the expected proportion of a source population that disperses from one location to another within a landscape [80]. For species of economic or environmental significance, this can prove useful in pest management initiatives. For instance, quantitative projections of the dispersal of recently introduced species invading new environments can support ‘slow the spread’ programmes [81].

Alternatively, spatial modeling can estimate the flow of organisms arriving at a defined area (“sink” habitat), by integrating the dispersal contribution from individuals originating from multiple “source” habitats within the surrounding landscape. In areas where suitable resources are locally abundant, individuals moving from numerous distant “source” habitats may combine to maintain high population densities in “sink” habitats [82]. However, because of the dilution effect in a two-dimensional space (i.e. a rapid decrease of the local density of dispersing organisms, with increasing distance), each distant source population will usually contribute few individuals to the flow of beetles arriving at a particular location.

In the forest landscape, woody resources suitable for pest species, such as bark beetles or wood borers, may become particularly abundant under particular circumstances, such as a severe storm, a fire, or following extensive thinning and clearcut harvesting operations. Large populations are there more likely to develop [83], hence potentially impacting the nearby forests by killing trees (aggressive species) or by affecting the quality of harvested wood (parasitic and saprophytic species). In New Zealand, large populations of the saprophytic bark beetles *Hylastes ater and Hylurgus ligniperda* are known to develop in managed *P*. *radiata* plantations on woody debris produced by thinning and clearcut harvesting. While dispersing, these can colonise freshly cut logs in nearby harvest areas, then become a phytosanitary concern for wood exports [28]. Spatial modeling is a prerequisite to identify zones within productive forests that may delineate an area of low pest prevalence [84]. Areas of low pest prevalence, either alone or in combination with other methods, such as a systems approach [85] represent a potential opportunity to tailor the application of pre-shipment treatments to mitigate the potential biosecurity risks of the commodity.

We show that at distances >1 km, the effects of dilution will spread dispersing bark beetles thinly across the landscape and it is likely that only particularly large or abundant sources may contribute to the flow of individuals arriving at a site. Hence, knowledge of dispersal distances and landscape based modelling can be used to identify zones within a managed production forest area where there is a low rate of arrival by dispersing beetles. Risk quantification in such zones provide options to store logs in areas of low pest prevalence, hence reducing the likelihood of insect colonization (as shown empirically by Mausel et al. [78]). Alternatively predicting the post-harvest exposure of logs to beetles within the environment contributes to an assessment of the phytosanitary risk that logs may pose to an importing country. Formal risk assessments of this nature can then be used to inform bilateral phytosanitary policies.

## Supporting information

S1 Appendix Summary of past mark-recapture experiments investigating dispersal in bark beetles. Table A. Published literature of mark-recapture experiments of bark beetles.
(DOCX)

S2 Appendix Description of traps used in the mark-release-recapture experiments. Fig A. Flight intercept panel trap used for mark-release-recapture experiments.
(DOCX)

S3 Appendix Weather conditions observed during the mark-release-recapture experiments. Fig A. Wind direction during the day of each release. Fig B. Wind speed during the day of each release. Fig C. Photosynthetic active radiation during the day of each release. Fig D. Air temperature during the day of each release. Fig E. Relative humidity during the day of each release.
(DOCX)

S4 Appendix Size-distance relationship. Fig A. Recaptured *Hylurgus ligniperda* size, measured as the width of the pronotum, as a function of distance of capture for male and female individuals. Fig B. Recaptured *Hylurgus ligniperda* size, measured as the width of the pronotum, as a function of distance of capture for flight-experienced and flight-naïve individuals.
(DOCX)

S5 Appendix Spatial location of captures. Fig A. Spatial location and number of marked *Hylurgus ligniperda* individuals recaptured in releases 1 to 5. Fig B. Spatial location and number of marked *Hylurgus ligniperda* individuals recaptured in releases 6 to 10. Fig C. Spatial location and number of marked *Hylurgus ligniperda* individuals recaptured in releases 11 to 15. Fig D. Spatial location and number of wild *Hylurgus ligniperda*, *Hylaster ater*, *Arhopalus ferus* and *Sirex noctilio* captured in releases 1 to 5. Fig E. Spatial location and number of wild *Hylurgus ligniperda*, *Hylaster ater*, *Arhopalus ferus* and *Sirex noctilio* captured in releases 6 to 11. Fig F. Spatial location and number of wild *Hylurgus ligniperda*, *Hylaster ater*, *Arhopalus ferus* and *Sirex noctilio* captured in releases 12 to 15.
(DOCX)
