# Supplementary material for: Quantifying dispersal of a non-aggressive saprophytic bark beetle
Source: PLoS One. 2017 Apr 13;12(4):e0174111. doi: 10.1371/journal.pone.0174111 (PMC5390978; doi:10.1371/journal.pone.0174111)
Supplement: S1 Appendix — Table A. Published literature of mark-recapture experiments of bark beetles. (DOCX) [file pone.0174111.s001.docx]

S1 Appendix. Summary of past mark-recapture experiments investigating dispersal in bark beetles.

In order to provide quantitative comparisons between our results and previous studies, we reviewed the published literature and summarized other mark-recapture experiments that investigated the dispersal ability of bark beetles (Table A). These studies addressed dispersal of primary, aggressive, (e.g. *Dendroctonus frontalis*) and secondary, opportunistically aggressive species (e.g. *Tomicus piniperda*). No previous studies of saprophytic bark beetles were found.

**Table A. Published literature of mark-recapture experiments of bark beetles.**

| **Species** | | **Hosts** | **Reference** | Released beetles | Traps | Lures | Max. distance |
| --- | --- | --- | --- | --- | --- | --- | --- |
| **Aggressive phloem feeders (high reliance on pheromones to perform mass attacks)** | | | | | | | |
|  | *Dendroctonus armandi* Tsai and Li | *Pinus* | [1] | Flight-naïve (collected from stripped trees) | Healthy *Pinus armandi* | None | 35 m |
|  | *Dendroctonus frontalis* Zimmermann | *Pinus* | [2] | Flight-naïve (emerging on site from logs) | Multi-funnels + lure | Pheromone + kairomone | 1,000 m |
|  | *Dendroctonus ponderosae* Hopkins | *Pinus* | [3] | Flight-naïve (emerging on site from logs) | Passive barriers | None | 200 m |
|  | *Dendroctonus pseudostugae* Hopkins | *Pinus* | [4] –expt 1 | Flight-naïve (emerging on site from logs) | Multi-funnels + lure | Pheromone + kairomone | 300 m |
|  |  |  | [4] –expt 2 | Flight-experienced (captured in pheromone traps) | Multi-funnels + lure | Pheromone + kairomone | 400 m |
|  | *Dendroctonus rufipennis* (Kirby) | *Picea* | [5] – “1991” expt | Flight-naïve (emerging on site from logs) | Multi-funnels + lure | Pheromone | 120 m |
|  |  |  | [5] – “1992” expt | Flight-naïve (emerging on site from logs) | Multi-funnels + lure | Pheromone | 600 m |
|  | *Ips grandicollis* (Eichoff) | *Pinus* | [6] | Flight-naïve (emerging in darkened cages from logs) | Multi-funnels + lure | Pheromone + kairomone | 2,000 |
|  | *Ips paraconfusus* (Say) | *Pinus* | [7] | Flight-naïve and flight-experienced at varying levels (after emerging in darkened room from logs or captured in traps) | Traps + pine bolts infested with males | Pheromone + kairomone | 2,000 |
|  | *Ips perturbatus* (Eichoff) | *Picea* | [5] | Flight-experienced (captured in pheromone traps) | Multi-funnels + lure | Pheromone | 120 m |
|  | *Ips sexdentatus* Boern | *Pinus* | [8] – expt 1 | Flight-naïve (collected from stripped trees or logs) | Barriers + lure | Pheromone + kairomone | 1,000 m |
|  |  |  | [8] – expt 2 | Flight-naïve (collected from stripped trees) | Barriers + lure | Pheromone + kairomone | 600 m |
|  | *Ips typographus* (L.) | *Picea* | [9] – expts 1 and 2 | Flight-experienced (captured in pheromone traps) | Barriers on healthy *Picea abies* + lure | Pheromone | 500 m |
|  |  |  | [9] – expt 3 | Flight-experienced (captured in pheromone traps) | Barriers on healthy *Picea abies* + lure | Pheromone | 750 m |
|  |  |  | [10] | Flight-naïve (emerging on site from infested trees) | Sticky barrier on healthy *Picea abies* | None | 30 m |
|  |  |  | [11] – “unflown” beetles | Flight-naïve (emerging on site from litter and bark) | Multi-pipes and funnels + lure | Pheromone | 50 m |
|  |  |  | [11] – “flown” beetles | Flight- experienced (captured in pheromone traps) | Multi-pipes and funnels + lure | Pheromone | 50 m |
|  |  |  | [12] – “1985” expt | Flight-naïve (emerging on site from litter and bark) | Multi-pipes and funnels + lure | Pheromone | 1,600 m |
|  |  |  | [12] – “1986” expt | Flight-naïve (emerging on site from litter and bark) | Multi-pipes and funnels + lure | Pheromone | 1,600 m |
|  |  |  | [13] – “1989” expt | Flight-experienced (1 day in insectary) | Barriers + lure | Pheromone | 1,000 m |
|  |  |  | [13] – “1990” expt | Flight-experienced (1 day in insectary) | Barriers + lure | Pheromone | 1,000 m |
|  |  |  | [14] – expt 2 | Flight-experienced (captured in pheromone traps) | Barriers + lure | Pheromone | 120 m |
|  |  |  | [15] – “flown beetles” expt | Flight-experienced (captured in pheromone traps) | Barriers + lure | Pheromone | 500 m |
|  |  |  | [15] – “unflown beetles” expt | Flight-naïve (emerging on site from logs) | Barriers + lure | Pheromone | 500 m |
|  |  |  | [16] – “non-baited trees” expt | Flight-naïve (emerging on site from bark) | Funnel on healthy *Picea abies* | Kairomone | 77 m |
|  |  |  | [16] – “baited tree” expt | Flight-naïve (emerging on site from bark) | Funnel on healthy *Picea abies* + lure | Pheromone | 35 m |
|  |  |  | [17] | Flight-naïve (emerging on site from bark) | Barriers on healthy *Picea abies* + lure | Pheromone | 258 m |
|  |  |  | [17] | Flight-naïve (emerging on site from bark) | Barriers on healthy *Picea abies* + lure | Pheromone | 339 m |
|  |  |  | [18] – “spring 2013” expt | Flight-naïve (emerging on site from infested logs) | Barriers + lure | Pheromone | 3,051 m |
|  |  |  | [18] – “summer 2013” expt | Flight-naïve (emerging on site from infested logs) | Barriers + lure | Pheromone | 3,051 m |
|  | *Scolytus multistriatus* (Marsham) | *Ulmus* | [19] – “1973” expt | Flight-naïve (emerging in darkened room from logs) | Traps + elm bolts infested with virgin females | Pheromone + kairomone | 402.5 m |
|  |  |  | [19] – “1975” expt | Flight-naïve and flight-experienced at varying levels (after emerging in darkened room from logs) | Sticky surfaces + lure | Pheromone | 305 m |
|  |  |  | [20] – “elms absent” expt | Flight-naïve (emerging on site from infested logs) | Sticky surfaces + lure | Pheromone + kairomone | 5,500 m |
| **Opportunistically aggressive phloem feeders (low reliance on pheromones to perform mass attacks)** | | | | | | | |
|  | *Dendroctonus valens* LeBonte | *Pinus* | [6] | Flight-experienced (captured in pheromone traps) | Multi-funnels + lure | Kairomone + pheromone | 2,000 |
|  | *Tomicus piniperda* (L.) | *Pinus* | [21] | Flight-naïve (emerging on site from infested logs) | Multi-funnels +lure, and logs | Kairomone | 400 m |
|  |  |  | [22] – “simulated mill yard” expt | Flight-naïve (emerging on site from infested logs) | Multi-funnels +lure, and logs | Kairomone | 100 m |
|  |  |  | [22] – “operational sawmill” expt | Flight-naïve (emerging on site from infested logs) | Multi-funnels +lure, and logs | Kairomone | 400 m |
| **Ambrosia beetles** | | | | | | | |
|  | *Gnathotrichus sulcatus* (LeConte) | Various conifers | [23] | Flight-experienced (captured in pheromone traps) | Sticky barriers + lure | Pheromone + kairomone | 1,300 m |
|  | *Trypodendron lineatum* (Olivier) | Various conifers | [23] | Flight-experienced (captured in pheromone traps) | Sticky barriers + lure | Pheromone + kairomone | 1,300 m |
|  |  |  | [24] – expt 1 | Flight-experienced (captured in pheromone traps) | Multi-funnels +lure | Pheromone | 50 m |
|  |  |  | [24] – expt 2 | Flight-experienced (captured in pheromone traps) | Multi-funnels +lure | Pheromone | 700 m |
|  |  |  | [24] – expt 3 | Flight-experienced (captured in pheromone traps) | Multi-funnels +lure | Pheromone | 1,000 m |
|  |  |  | [24] – expt 4 | Flight-experienced (captured in pheromone traps) | Multi-funnels +lure | Pheromone | 1,900 m |
|  |  |  | [24] – expt 5 | Flight-experienced (captured in pheromone traps) | Multi-funnels +lure | Pheromone | 1,900 m |
|  |  |  | [25] – expt 1 | Flight-experienced (captured in pheromone traps) | Multi-funnels +lure | Pheromone + kairomone | 1,830 m |
|  |  |  | [25] – expt 2 | Flight-experienced (captured in pheromone traps) | Multi-funnels +lure | Pheromone + kairomone | 1,830 m |

Quantitative data from references [2, 4, 6-8, 13, 15, 19, 20, 22, 23] are discussed in the article.

We compared the capture rates of *Hylurgus ligniperda* release-recapture experiments and estimated proportion of beetles settling at given distances with results from the studies listed in the Table above. Below we provide a short summary of the mark-recapture protocol for these studies in which we have extracted estimates of capture rates per trap at specific distances. These were extracted directly from the text, or from inspection of the Tables and Figures.

[2] performed eight spatially and temporally replicated release-recaptures of the southern pine beetle *Dendroctonus frontalis* Zimmermann. Experiments were performed in mature pine stands in Louisiana, USA, in autumn 1989 and spring-summer 1990. Flight-naïve individuals emerged from bolts containing brood and marked by fluorescent dust during the emergence process. Considering only the replicates for which the number of emerging beetles had been estimated from the average beetle density on a subsample (i.e. 1-2, 1-3, 1-4, 2-1, 2-2, 4-1, 4-2 and 4-3), 362,600 beetles were released (estimates from Table 1 in [2]). These were all considered flyers. Marked beetles were captured in a network of 46 traps located in concentric rings around a single release point, with radii 50, 100, 200, 400, 600, 800 and 1,000m (2, 4, 8, 8, 8, 8 and 8 traps). Traps were funnel traps [26] baited with frontalin and natural turpentine. For the considered replicates, 1,750 beetles (0.010% per trap) were recaptured in the entire network (estimates from Table 1 in [2]), with 158 individuals (0.022% per trap) at the nearest distance of 50 m, and 52 individuals (0.0018% per trap) at the furthest distance of 1,000 m (estimates from Figure 3 in [2]).

[4] performed six spatially and temporally replicated release-recaptures of the Douglas-fir beetle *Dendroctonus pseudotsugae* Hopkins. Experiments were performed in mixed clearings, regeneration and mature stands of ponderosa pines, Idaho, USA. In spring 2000, a total of 5,385 flight-naïve beetles emerging from bolts containing brood were marked by fluorescent dust during the emergence process and released (estimates from Table 3 in [4]). Multiple releases were performed on transects NS and WE centered on one trap (50, 100, 200 and 300 m from trap). In spring 2000 and 2001, a total of 1,468 flight-experienced individuals previously captured in pheromone and kairomone baited pipe traps, were marked by fluorescent dust and released (estimates from Table 4 in [4]). Multiple releases were performed on one transect leading away from one trap (50, 100, 200, 300 and 400 m from trap). All released beetles were considered flyers. Traps were 16-unit multiple-funnels trap baited with frontalin, seudenol and ethanol. 302 flight-naïve beetles (5.6% per trap) were recaptured in the entire network, with 84 individuals (6.2% per trap) at the nearest distance of 50 m, and 41 individuals (2.9% per trap) at the furthest distance of 300 m (estimates from Table 3 in [4]). 62 flight-experienced beetles (4.2% per trap) were recaptured in the entire network, with 18 individuals (6.2% per trap) at the nearest distance of 50 m, and 7 individuals (1.0% per trap) at the furthest distance of 400 m (estimates from Table 4 in [4]).

[7] performed 9 temporally replicated release-recaptures of the five-spined engraver *Ips paraconfusus* Lanier (second series of tests on physiological conditions in [7]). Experiments were performed in a stand of second-growth ponderosa pine in California, USA, in summer 1962. A total of 6,988 flight-naïve beetles emerging from caged bolts containing brood, and a total of 9,218 flight-experienced beetles previously captured in field olfactometers were marked by fluorescent dust (estimates from Table V in [7]). Beetles were released in subsequently set up circles of 5 traps around a single release point, with radii 3, 5, 10, 25, 50, 100, 500, 1000 and 2,000m. Traps were funnel-based olfactometerss baited with log sections of ponderosa pine, each infested with 25 males. 991 flight-naïve beetles (2.8% per trap) were recaptured in the entire network, with 286 individuals (5.6% per trap) at the nearest distance of 5 m, 28 individuals (1.2% per trap) at 50 m, and 2 individuals (0.14% per trap) at 1.000 m (estimates from Table V in [7]). 1,404 flight-experienced beetles (3.0% per trap) were recaptured in the entire network, with 364 individuals (5.3% per trap) at the nearest distance of 5 m, 156 individuals (3.3% per trap) at 50 m and 9 individuals (0.26% per trap) at 1,000 m (estimates from Table V in [7]).

[8] performed 27 spatially and temporally replicated release-recaptures of the six-spined engraver beetle *Ips sexdentatus* (Börner). Experiments were performed in mature Scots pine forest in southwestern France, in summer 1989 and summer 1990. Flight-naïve individuals were obtained from trap trees and laboratory breedings. They were marked by elytral engraving before being released from a platform in the centre of the study site. Considering only experiment 1 and replicates 2 and 3 for which the largest range of distances had been estimated (5 plots per replicate), 3,995 beetles were released (estimates from Table I in [8]) and 3,784 (94.7%) of these were considered flyers. Marked beetles were captured in a network of 20 traps located in concentric rings around a single release point, with radii 50, 100, 200, 500 or 1000 m (4 traps each). Traps were barrier-traps with flat funnels (Röchling model) baited with a mixture of methyl butenol, ipsdienol and α-pinene. From the considered replicates, 1,037 beetles (1.37% per trap) were recaptured in the entire network, with 222 individuals (1.47% per trap) at the nearest distance of 50 m, and 74 individuals (0.49% per trap) at the furthest distance of 1,000 m (estimates from Figure 1 in [8]).

[13] performed five temporally replicated release-recaptures of the spruce bark beetle *Ips typographus* L. experiments were performed in 50 to 110-year-old spruce forest in Southern Bohemia (former Czechoslovakia), in spring 1989 and spring 1990. Individuals reared in an insectary were kept at 22-24˚C with flight activity regime for 24 hours, then caught and marked by fluorescent dust, and released at a single release point. 3,800 beetles were released in 1989 and 2,800 in 1990 (Table in [13]) and all of these were considered flyers. Marked beetles were recaptured in a network of 44 groups of 4 traps located in concentric rings around the release point, with radii 50, 100, 200, 300, 400, 500, 600, 700, 800, 900 and 1,000 m (4 traps at each distance). Traps were baited with pheromone (Pheroprax). 1,033 and 458 marked beetles (0.62% and 0.36% per trap) were recaptured in the entire network in 1989 and 1990, respectively (Table in [13]). 397 (2.61% per trap) and 196 individuals (1.75% per trap) were recaptured at the nearest distance of 50 m, (1989 and 1990 estimates, respectively, from Figure 2 in [13]). 29 (0.19 % per trap) and 16 individuals (0.147 % per trap) were recaptured at the furthest distance of 1,000 m (1989 and 1990 estimates, respectively, proportions given in the text).

[19] performed three temporal release-recapture of the smaller European elm bark beetle *Scolytus multistriatus* (Marsham) in a farm area north of Delaware, Ohio, in summer 1975.

Individuals emerging from bolts initially containing brood in a darkened rearing were exposed to five conditioning regimes: (1) food and flight exercise for 24hr; (2) food, but no flight exercise of 24 hr; (3) flight exercise, but no food, for 24 hr; (4) 24 hr with no food or flight exercise; and (5) no time lapse after emergence, no food or flight exercise. Beetles were released in the center of a grid of 64 traps spaced at 46 m, later modified to a 52 traps grid. Traps were 2 dimensional sticky traps baited with cubebene, multistriatine and heptanol. Flight and captures in the “no treatment” regime were superior to all other regimes. There 99% of the 9,767 beetles released flew and 4.7 to 16.4% of them were recaptured, depending on the release day (estimates from Table 2 in [19]). In contrast, 67-75% of the 8,600 beetles released in the “flight 24 hr” regime flew and 2.0-5.1% of them were recaptured, while 50-87% of the 7,975 beetles released in the “held 24hr” regime flew and only 0.6-3.6% of them were recaptured. Most captured were performed at 32 m, the shortest distance from release, 243 beetles in the “no treatment” regime (0.48% per trap), 54 in the “flight 24 hr” regime (0.083% per trap) and 5 in the “held 24 hr” regime (0.025% per trap). At larger distances, recapture rates are still higher in the “no treatment” regime, but differences between the “flight 24 hr” and the “held 24 hr” regimes tend to disappear.

[20] performed one release-recapture of the smaller European elm bark beetle *Scolytus multistriatus* (Marsham) in a predominantly bush/scrub covered location in Eureka Valley, California, in summer 1979. The valley is isolated with no elms and surrounded by mountains of 3,000 m elevation, with the nearest elms being a few individuals located 14 km away but separated from the study site by mountain ridges. Flight-naïve individuals from bolts initially containing brood or callow adults were released at a single release point. 20,924 beetles were estimated to have emerged from these logs and were considered flyers. Marked beetles were recaptured in a network of 56 traps located 150-5,500 m away from the release point. Traps were sticky paper traps baited with cubebene, multistriatine and heptanol. 48 of the traps were stapled near the upper end of 2 m cardboard tubes and 8 of them were fixed to 2 m long elm logs. 231 beetles (0.02% per trap) were recaptured in the entire network, with 79 individuals (0.094% per trap) at the nearest distances of 150 m, and 3 individuals (0.007% per trap) in the two traps at 1500 m (estimates from Figure 2 in [20]). None were captured in the two traps located at 5500 m.

[22] performed 4 (expt 1) and 5 (expt 2) spatial replicates release-recapture of the pine shoot beetle *Tomicus piniperda* (L.). Experiments 1 and 2 were performed in an open agricultural field, and in an operation sawmill, respectively, in Michigan, in spring 1998.

Flight-naïve beetles emerging from bolts containing brood were marked by fluorescent dust during the emergence process and released in a network traps located in concentric rings around the release point, with radii 25, 50 and 100 m (4 traps at each distance) in expt 1, and 0-20 m (9 traps), 50, 100, 200 and 400 m (4 traps at each distance) in expt 2. Traps in expt 1 (simulated sawmill) were composed of three uninfested red pine logs and one 12-unit multiple funnel trap baited with alpha-pinene. Traps in expt 2 (operational sawmill) were composed 12-unit multiple funnel traps baited with alpha-pinene. In expt 1, 3 of a total of 1661 released beetles (0.015% per trap) have been recaptured, with 2 individuals (0.030% per trap) at the nearest tested distance of 25 m, and 1 individual (0.015% per trap) at 50 m, which is the furthest distance at which captures occurred (estimates from Table 2 in [22]). In expt 2, 19 of a total of 2117 released beetles (0.040% per trap) have been recaptured, with 8 individuals (0.042% per trap) in the immediate vicinity of the release point (<25m), 2 individuals (0.026% per trap) at 50 m, and 4 individuals (0.056% per trap) in the 200-250 m range, which is the furthest distance at which captures occurred (estimates from Table 2 in [22]).

[23] performed one release-recapture of the striped ambrosia beetle *Trypodendron lineatum* (Olivier). Experiment was performed in a saw mill, in southern British Columbia, in spring 1981. Flight-experienced individuals were captured in pheromone and kairomone baited pipe traps, marked by fluorescent dust, and then released at a single release point. 6,000 beetles were released and 3,400 (57%) of these were considered flyers. Marked beetles were recaptured in a network of 14 “suppression”, traps located 30-1,300 m away from the release point. Traps were sticky panel traps baited with lineatin, sulcatol, α-pinene and ethanol. 202 beetles (0.42% per trap) were recaptured in the entire network, with 119 individuals (0.16% per trap) at the nearest distances of 30-200 m, and 21 individuals (0.018% per trap) at the furthest distances of 500–1,300 m (estimates from Table 2 in [23]).

References

1. Wang X, Chen H, Ma C, Li Z. Chinese white pine beetle, *Dendroctonus armandi* (Coleoptera: Scolytinae), population density and dispersal estimated by mark-release-recapture in Qinling Mountains, Shaanxi, China. Appl Entomol Zoo. 2010. 45:557–567.
2. Turchin P, Thoeny WT. Quantifying dispersal of Southern pine beetles with mark-recapture experiments and a diffusion model. Ecol Appl. 1993; 3:187–198.
3. Safranyik L, Silversides R, McMullen LH, Linton DA. An empirical approach to modeling the local dispersal of the mountain pine beetle (*Dendroctonus ponderosae* Hopk.) (Col., Scolytidae) in relation to sources of attraction, wind direction and speed. J Appl Entomol. 1989; 108 498–511.
4. Dodds KJ, Ross DW. Sampling range and range of attraction of *Dendroctonus pseudotsugae* pheromone-baited traps. Can Entomol. 2002; 134:343–355.
5. Werner RA, Holsten EH. Dispersal of the spruce beetle, *Dendroctonus rufipennis*, and the engraver beetle, *Ips perturbatus* in Alaska. USDA Forest Service Pacific Northwest Research Station Research Paper. 1997.
6. Costa A, Min A, Boone CK, Kendrick AP, Murphy RJ, Sharpee WC, Raffa KF, Reeve JD. Dispersal and edge behaviour of bark beetles and predators inhabiting red pine plantations. Agr Forest Entomol. 2013; 15:1–11.
7. Gara RI. Studies on the flight behavior of *Ips confusus* (LeC.) (Coleoptera: Scolytidae) in response to attractive material. Contrib Boyce Thomps. 1963; 22:51–66.
8. Jactel H. Dispersal and flight behavior of *Ips sexdentatus* (Coleoptera, Scolytidae) in pine forest. Ann For Sci. 1991; 48:417–428.
9. Botterweg PF. Dispersal and flight behaviour of the spruce bark beetle *Ips typographus* in relation to sex, size and fat content. Z Angew Entomol. 1982; 94: 466–489.
10. Anderbrant O. Dispersal of reemerged spruce bark beetles, *Ips typographus* (Coleoptera, Scolytidae): a mark-recapture experiment. Z Angew Entomol. 1985; 99:21–25.
11. Weslien J, Lindelöw Å. Trapping a local population of spruce bark beetles *Ips typographus* (L.): Population size and origin of trapped beetles. Ecography. 1989; 12:511–514.
12. Weslien J, Lindelöw Å. Recapture of marked spruce bark beetles (*Ips typographus*) in pheromone traps using area-wide mass trapping. Can J Forest Res. 1990; 20:1786–1790.
13. Zumr V. Dispersal of the spruce bark beetle *Ips typographus* (L.) (Col., Scolytidae) in spruce woods. J Appl Entomol. 1992; 114:348–352.
14. Zolubas P, Byers JA. Recapture of dispersing bark beetle *Ips typographus* (L.) (Col., Scolytidae) in pheromone-baited traps: Regression models. J Appl Entomol. 1995; 119:285–289.
15. Duelli P, Zahradnik P, Knizek M, Kalinova B. Migration in spruce bark beetles (*Ips typographus* L.) and the efficiency of pheromone traps. J Appl Entomol. 1997; 121:297–303.
16. Franklin AJ, Grégoire J-C. Flight behaviour of *Ips typographus* L. (Col., Scolytidae) in an environment without pheromones. Ann For Sci. 1999; 56:591–598.
17. Franklin AJ, Debruyne C, Grégoire J-C. Recapture of *Ips typographus* L. (Col., Scolytidae) with attractants of low release rates: localized dispersion and environmental influences. Agr Forest Entomol. 2000; 2:259–270.
18. Doležal P, Okrouhlík J, Davídková M. Fine fluorescent powder marking study of dispersal in the spruce bark beetle, *Ips typographus* (Coleoptera: Scolytidae). Eur J Entomol. 2016; 113: 1–8.
19. Wollerman EH. Attraction of European elm bark beetles, *Scolytus multistriatus*, to pheromone-baited traps. J Chem Ecol. 1979; 5:781–793.
20. Birch MC, Miller JC, Paine TD. Evaluation of two attempts to trap defined populations of *Scolytus multistriatus*. J Chem Ecol. 1982. 8:125–136.
21. Barak AV, McGrevy D, Tokaya G. Dispersal and re-capture of marked, overwintering *Tomicus piniperda* (Coleoptera: Scolytidae) from Scotch pine bolts. Great Lakes Entomol. 2000; 33:69–80.
22. Poland TM, Haack RA, Petrice TR, Sadof CS, Onstad DW. Dispersal of *Tomicus piniperda* (Coleoptera: Scolytidae) from operational and simulated mill yards. Can. Entomol. 2000; 132:853–866.
23. Shore TL, McLean JA. The use of mark-recapture to evaluate a pheromone-based mass trapping program for ambrosia beetles in a sawmill. Can J Forest Res. 1988; 18:1113–1117.
24. Salom SM, McLean JA. Dispersal of *Trypodendron lineatum* (Olivier) within a valley setting. Can Entomol. 1990; 122:43–58.
25. Salom SM, McLean JA. Environmental influences on dispersal of *Trypodendron lineatum* (Coleoptera: Scolytidae). Environ Entomol. 1991; 20:565–576.
26. Lindgren BS. A multiple funnel trap for scolytid beetles (Coleoptera). Can Entomol. 1983. 115:299–302.
