# Supplementary material for: Quantifying dispersal of a non-aggressive saprophytic bark beetle
Source: PLoS One. 2017 Apr 13;12(4):e0174111. doi: 10.1371/journal.pone.0174111 (PMC5390978; doi:10.1371/journal.pone.0174111)
Supplement: S2 Appendix — Fig A. Flight intercept panel trap used for mark-release-recapture experiments. (DOCX) [file pone.0174111.s002.docx]

S2 Appendix. Description of traps used in the mark-release-recapture experiments.

Traps used in this study were custom-made four-vane black panel traps, suspended from 1.6m steel posts baited with a single 150 mL of 95% alpha-pinene-filled and a single 150 mL 98% ethanol-filled sealed 150 μm thick polyethylene dispenser (Fig A).


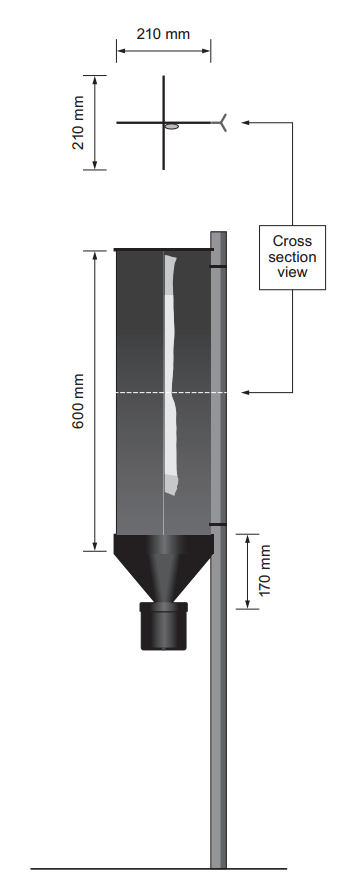


**Fig A. Flight intercept panel trap used for mark-release-recapture experiments.**
