# Supplementary material for: Quantifying dispersal of a non-aggressive saprophytic bark beetle
Source: PLoS One. 2017 Apr 13;12(4):e0174111. doi: 10.1371/journal.pone.0174111 (PMC5390978; doi:10.1371/journal.pone.0174111)
Supplement: S3 Appendix — Fig A. Wind direction during the day of each release. Fig B. Wind speed during the day of each release. Fig C. Photosynthetic active radiation during the day of each release. Fig D. Air temperature during the day of each release. Fig E. Relative humidity during the day of each release. (DOCX) [file pone.0174111.s003.docx]

S3 Appendix. Weather conditions observed during the mark-release-recapture experiments.

Meteorological data was collected at the location indicated in Fig 1 using a 2.5 m metal tower (Scottech, Hamilton, New Zealand). Data from sensors was recorded on a CR1000 (Campbell Scientific, Logan, USA) data logger with measurements taken every minute. Sensors included a RM Young wind monitor (model 05103, RM Young Company, Michigan, USA); Apogee quantum sun calibration sensor (model sq-110 photosynthetic radiation sensor, Apogee Instruments, Logan, USA); CSI temperature and relative humidity probe (model hc2s3, Campbell Scientific, Logan, USA); CSI rain gauge (model tb4, Campbell Scientific, Logan, USA).

Hourly averages are reported below for wind direction (Fig A), wind speed (Fig B), photosynthetically active radiation (Fig C), air temperature (Fig D) and relative humidity (Fig E). Rainfall has not been observed during the releases and is not reported. Dark grey shaded areas on the left and right sides of the graphs indicate hours before sunrise and after sunset, respectively. A shift due to daylight saving occurred after 6 April hence the large shift in the dark shaded area at release 10. Light grey shaded areas indicate the temporal extent of the observed beetles take-offs from the release platform, for each release. Measurements have not been collected for releases 3, 4 and part of release 5 due to recording failure of the data logger.


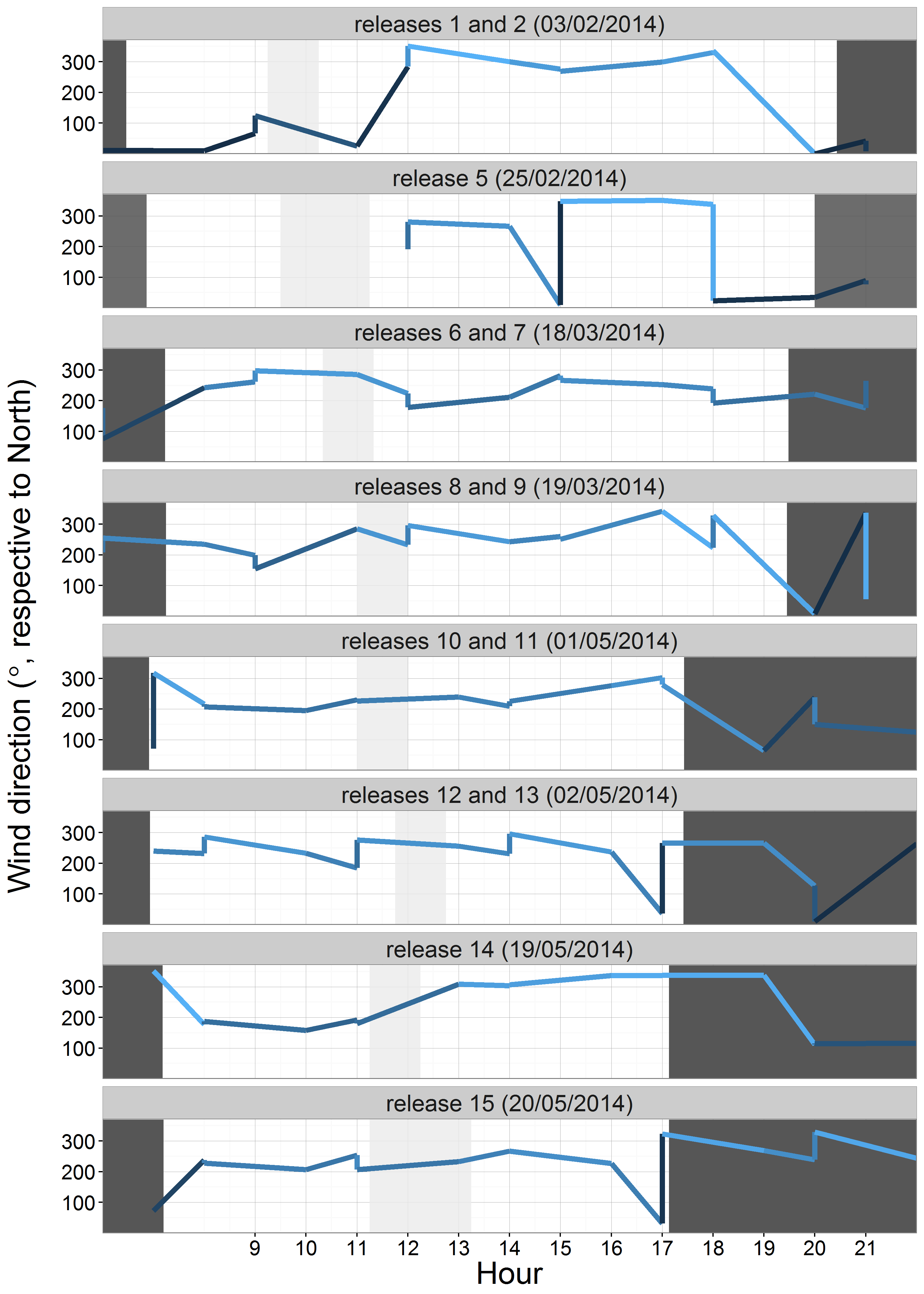


**Fig A. Wind direction during the day of each release.
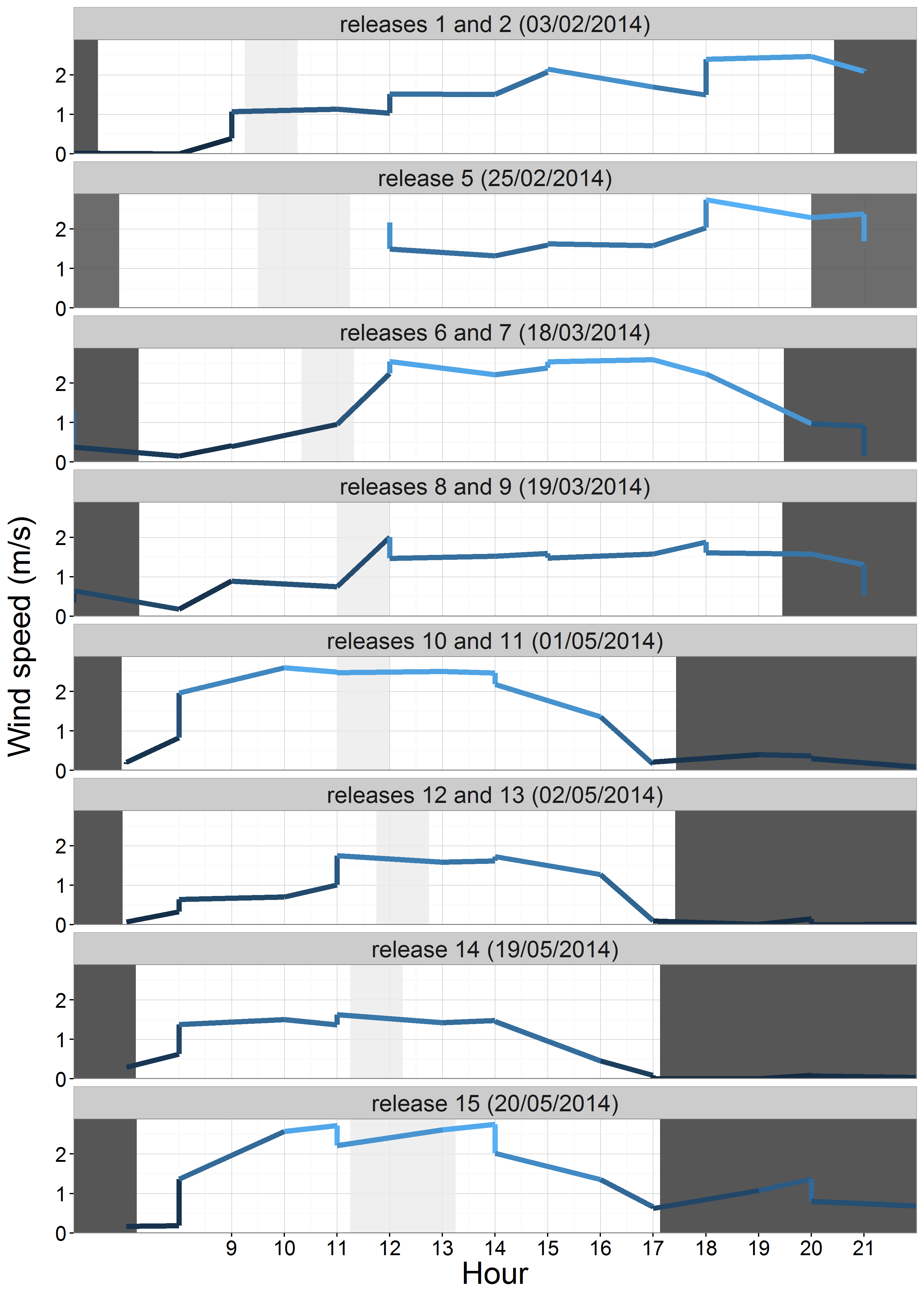
**

**Fig B. Wind speed during the day of each release.**


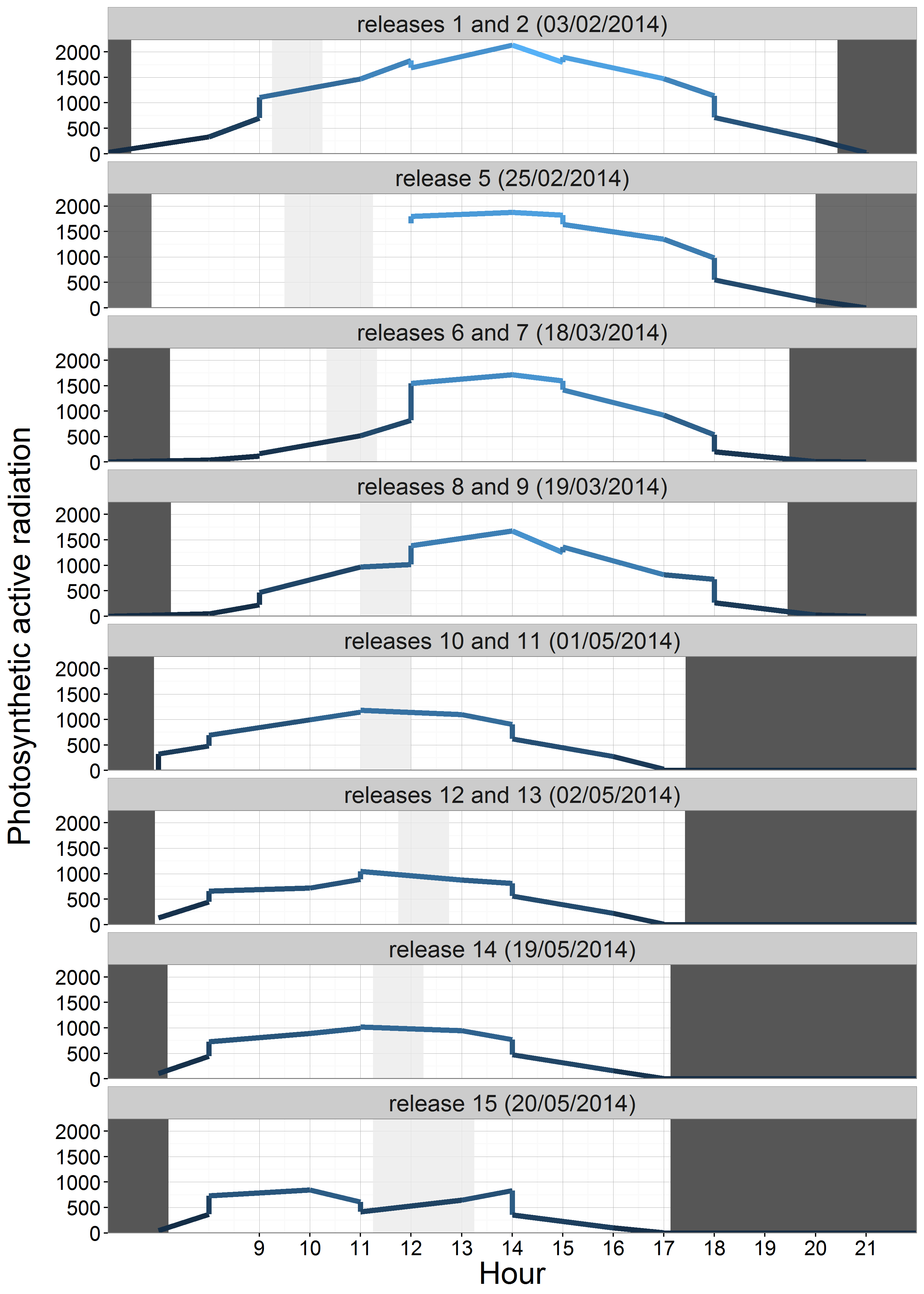


**Fig C. Photosynthetic active radiation during the day of each release.**


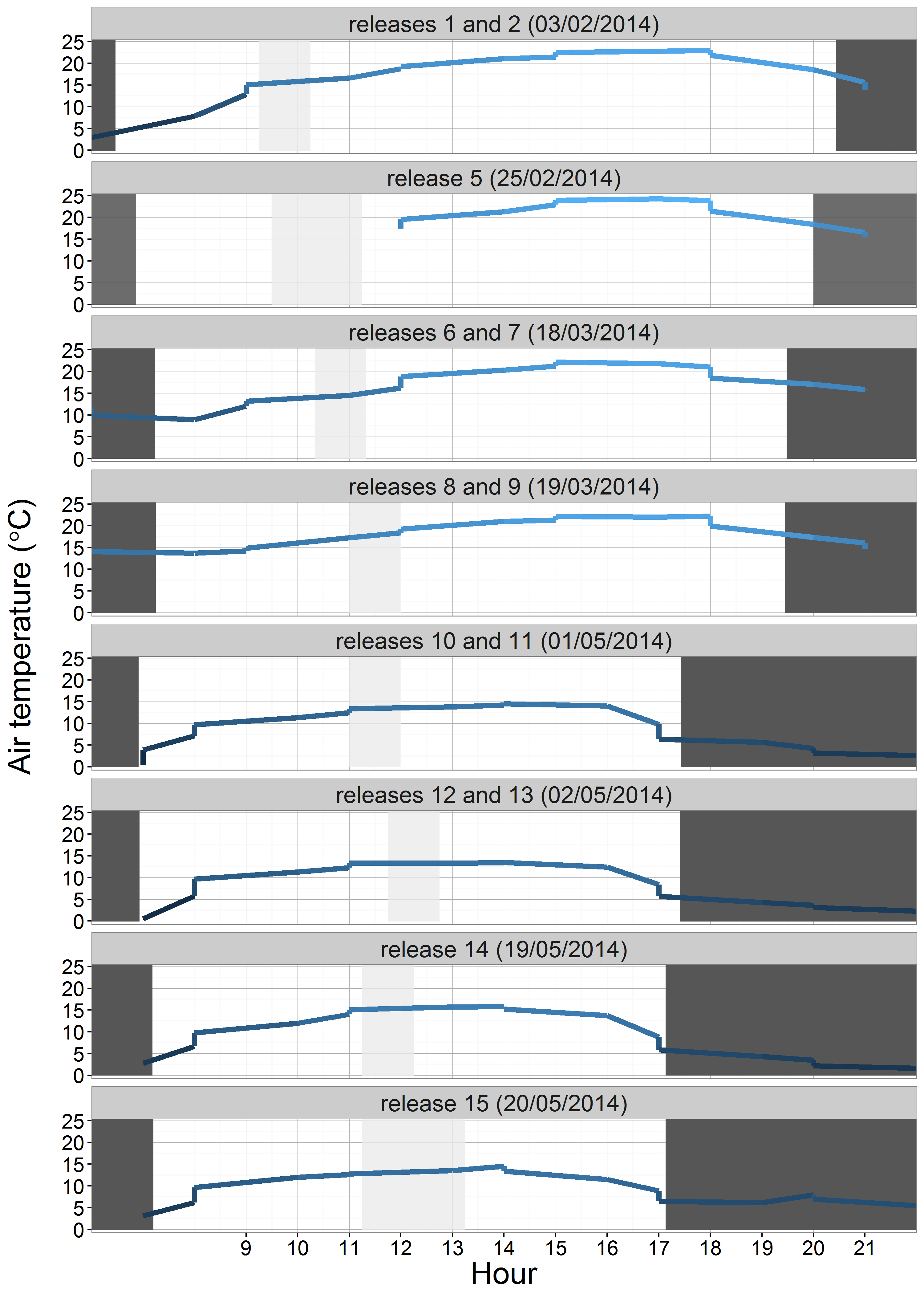


**Fig D. Air temperature during the day of each release.**


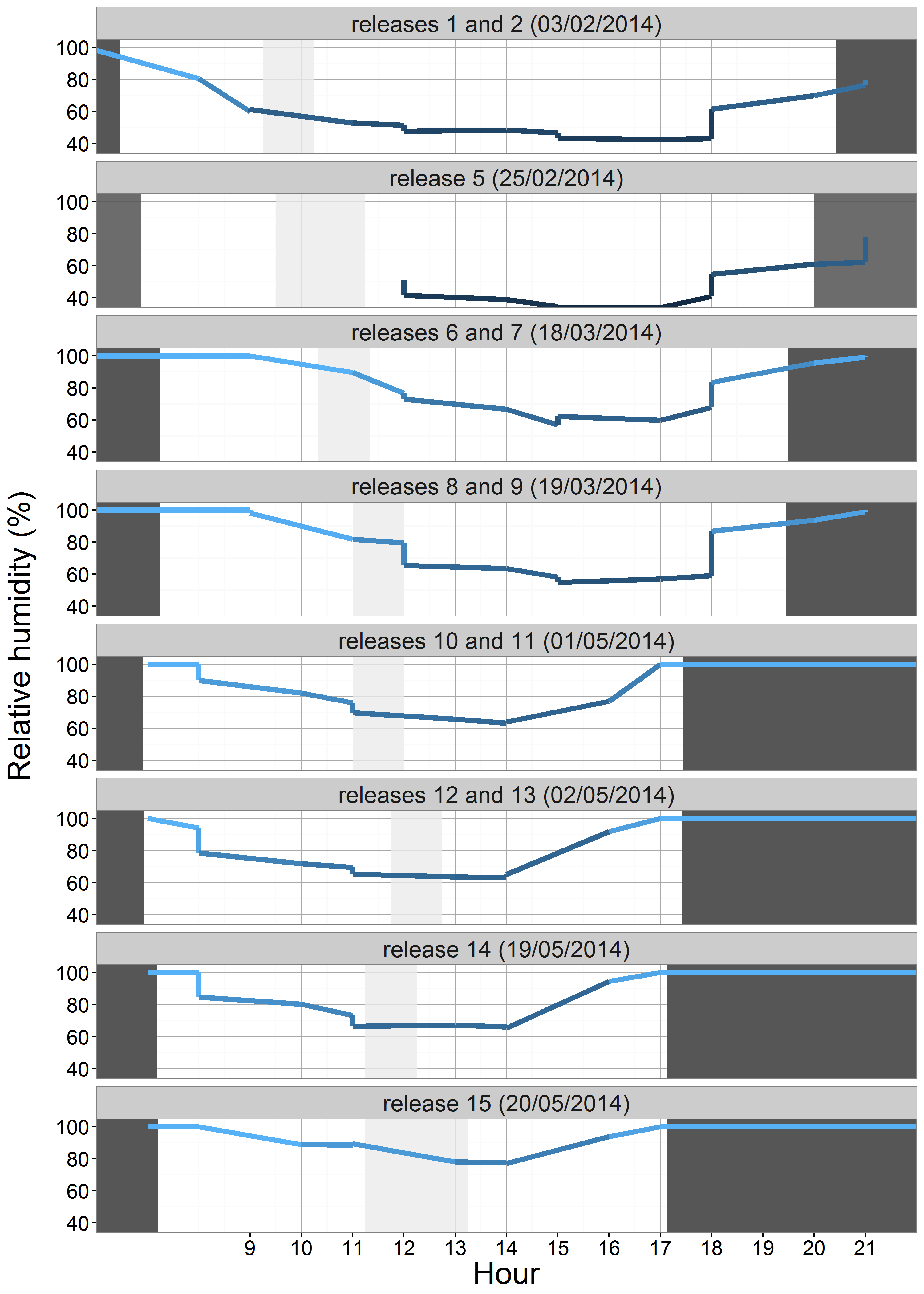


**Fig E. Relative humidity during the day of each release.**
