# Supplementary material for: Quantifying dispersal of a non-aggressive saprophytic bark beetle
Source: PLoS One. 2017 Apr 13;12(4):e0174111. doi: 10.1371/journal.pone.0174111 (PMC5390978; doi:10.1371/journal.pone.0174111)
Supplement: S4 Appendix — Fig A. Recaptured Hylurgus ligniperda size, measured as the width of the pronotum, as a function of distance of capture for male and female individuals. Fig B. Recaptured Hylurgus ligniperda size, measured as the width of the pronotum, as a function of distance of capture for flight-experienced and flight-naïve individuals. (DOCX) [file pone.0174111.s004.docx]

S4 Appendix. Size-distance relationship

**Fig A. Recaptured *Hylurgus ligniperda* size, measured as the width of the pronotum, as a function of distance of capture for male and female individuals.** Generalized additive models with integrated smoothness estimation. Grey shaded area corresponds to 95% confidence intervals. A small amount of jitter was applied to the data points so overlapping records can be distinguished. Note that the x-axis is on a log 10 scale.


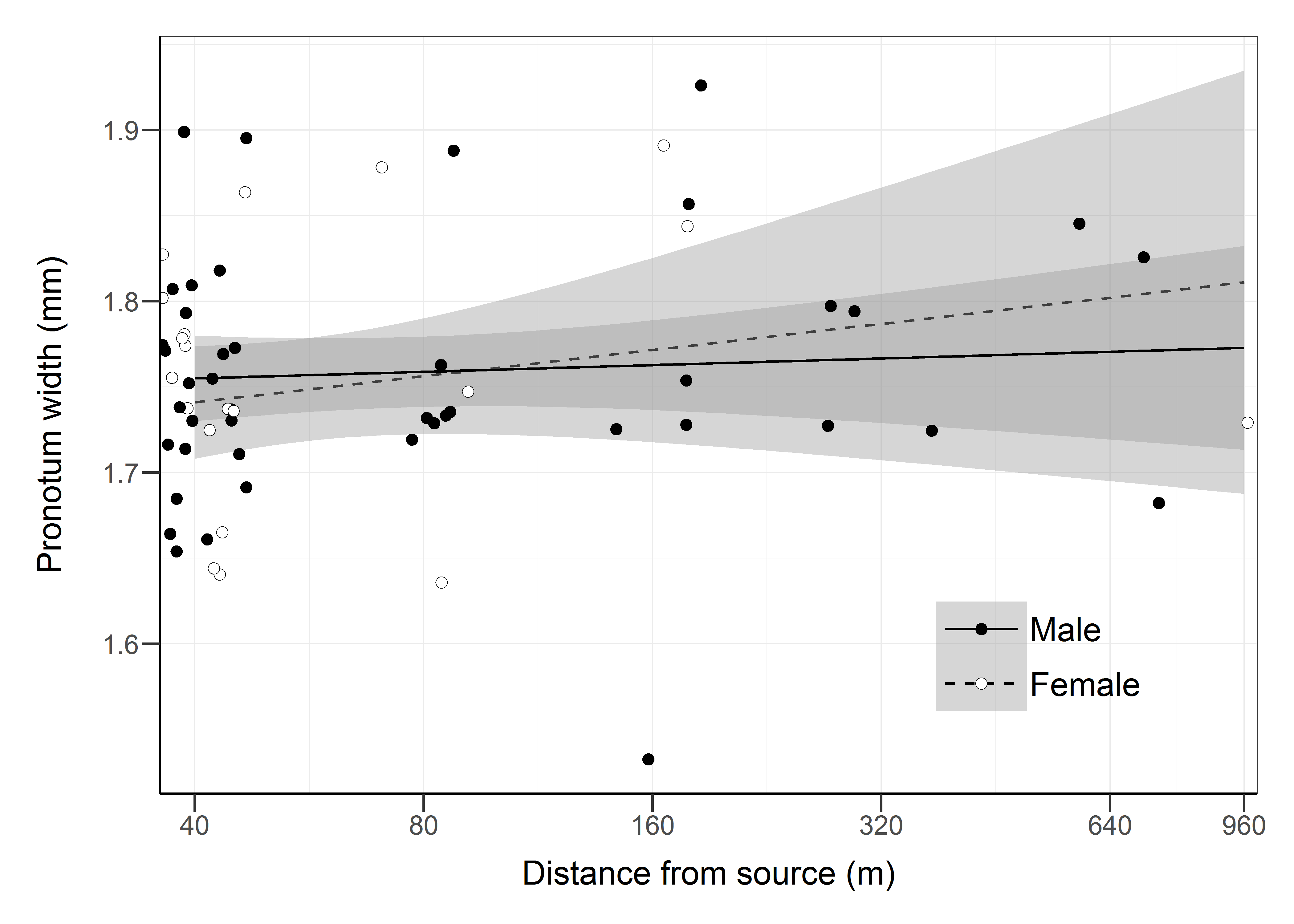


**
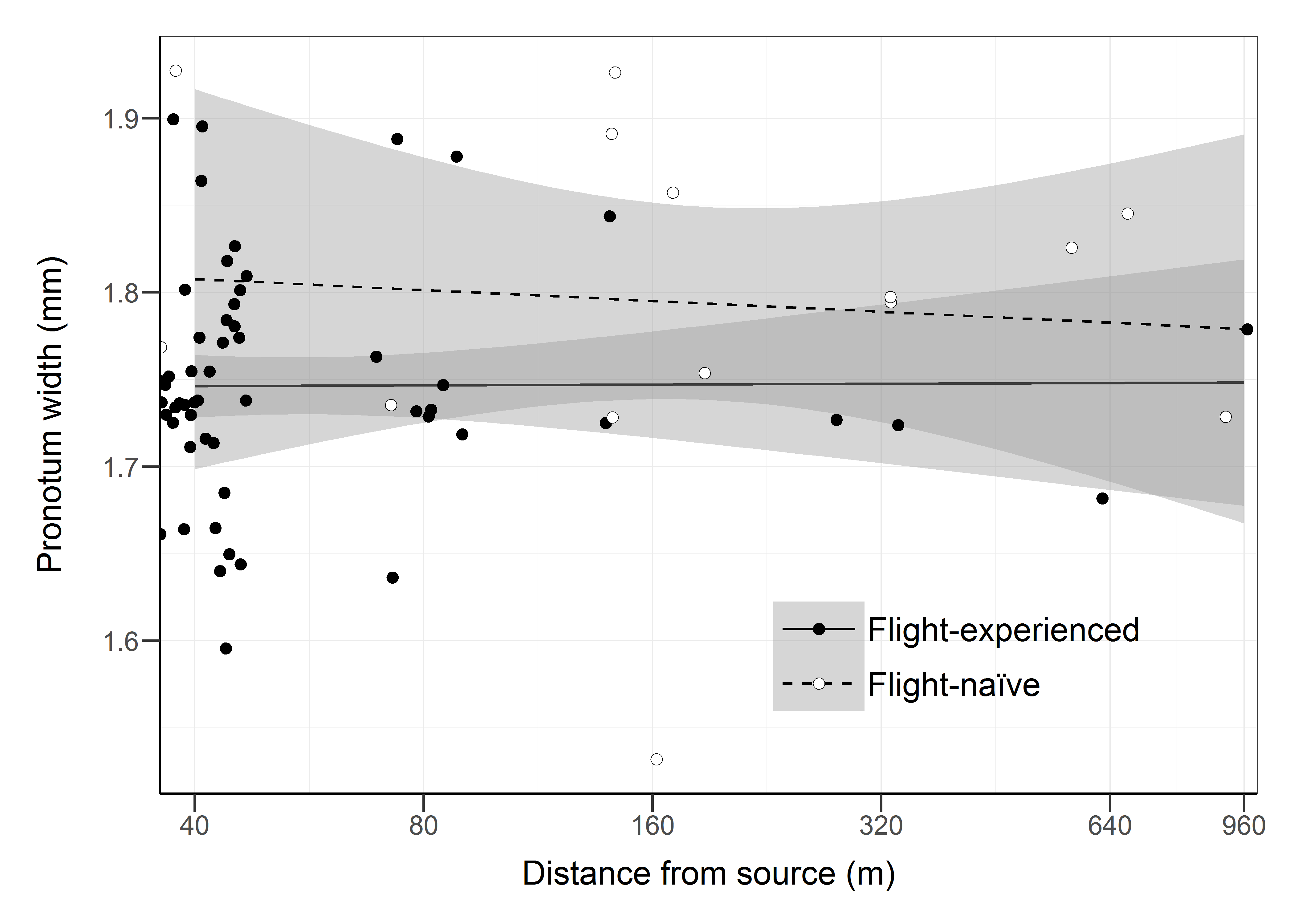
**

**Fig B. Recaptured *Hylurgus ligniperda* size, measured as the width of the pronotum, as a function of distance of capture for flight-experienced and flight-naïve individuals.** Generalized additive models with integrated smoothness estimation. Grey shaded area corresponds to 95% confidence intervals. A small amount of jitter was applied to the data points so overlapping records can be distinguished. Note that the x-axis is on a log 10 scale.
