# Supplementary material for: Quantifying dispersal of a non-aggressive saprophytic bark beetle
Source: PLoS One. 2017 Apr 13;12(4):e0174111. doi: 10.1371/journal.pone.0174111 (PMC5390978; doi:10.1371/journal.pone.0174111)
Supplement: S5 Appendix — Fig A. Spatial location and number of marked Hylurgus ligniperda individuals recaptured in releases 1 to 5. Fig B. Spatial location and number of marked Hylurgus ligniperda individuals recaptured in releases 6 to 10. Fig C. Spatial location and number of marked Hylurgus ligniperda individuals recaptured in releases 11 to 15. Fig D. Spatial location and number of wild Hylurgus ligniperda, Hylaster ater, Arhopalus ferus and Sirex noctilio captured in releases 1 to 5. Fig E. Spatial location and number of wild Hylurgus ligniperda, Hylaster ater, Arhopalus ferus and Sirex noctilio captured in releases 6 to 11. Fig F. Spatial location and number of wild Hylurgus ligniperda, Hylaster ater, Arhopalus ferus and Sirex noctilio captured in releases 12 to 15. (DOCX) [file pone.0174111.s005.docx]

S5 Appendix. Spatial location of captures.

The spatial location and number of individuals captured are indicated for marked *Hylurgus ligniperda* (Figs A-C), and for the four species of wild bark beetles and wood borers that have also been regularly captured in the traps, *Hylurgus ligniperda* (Fabricius) and *Hylastes ater* (Paykull) (Coleoptera: Scolytinae), *Arhopalus ferus* (Mulsant) (Coleoptera: Cerambycidae), and *Sirex noctilio* Fabricius (Hymenoptera: Siricidae) (Figs D-F)*.*

**
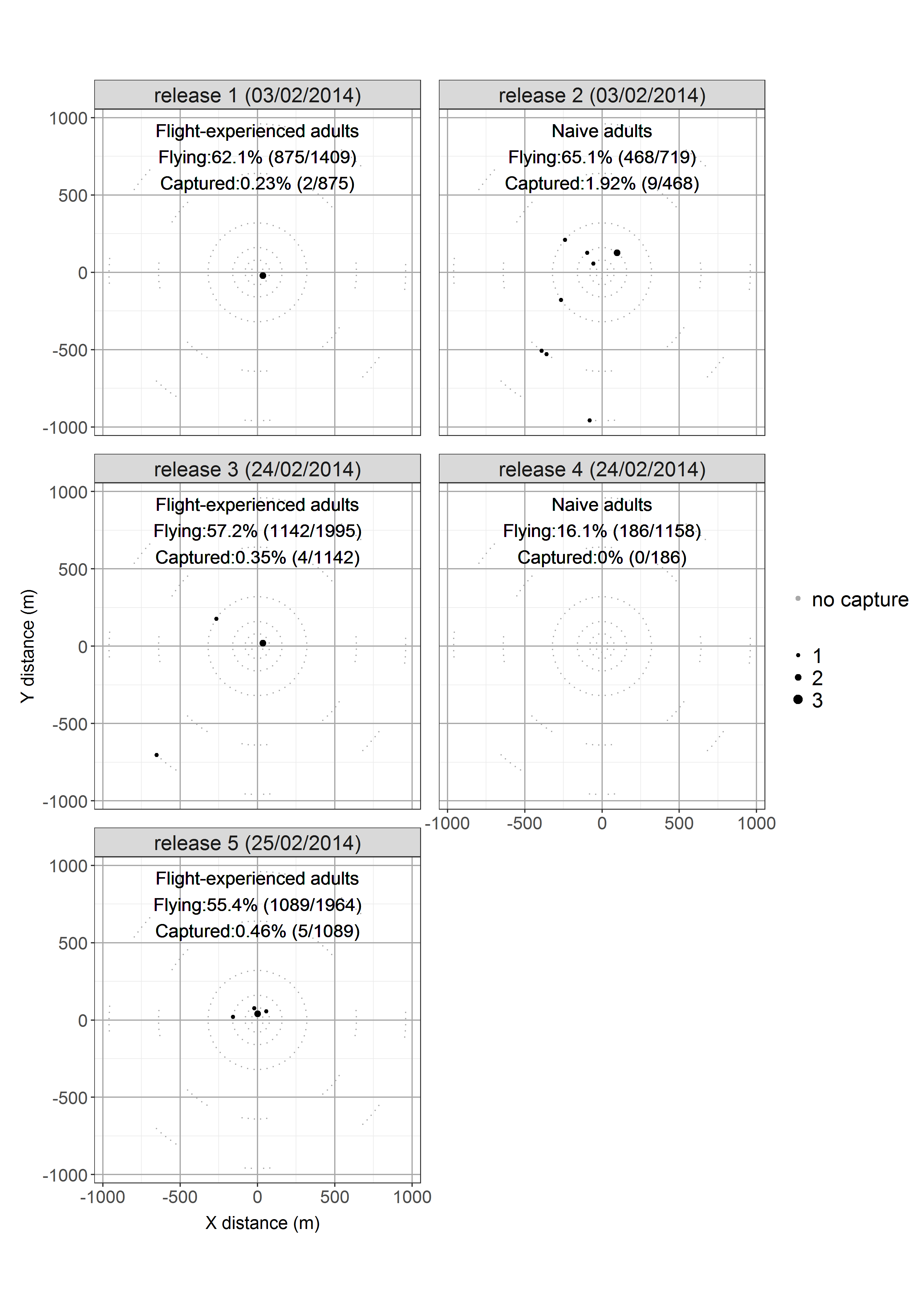
**

**Fig A. Spatial location and number of marked *Hylurgus ligniperda* individuals recaptured in releases 1 to 5.**

**
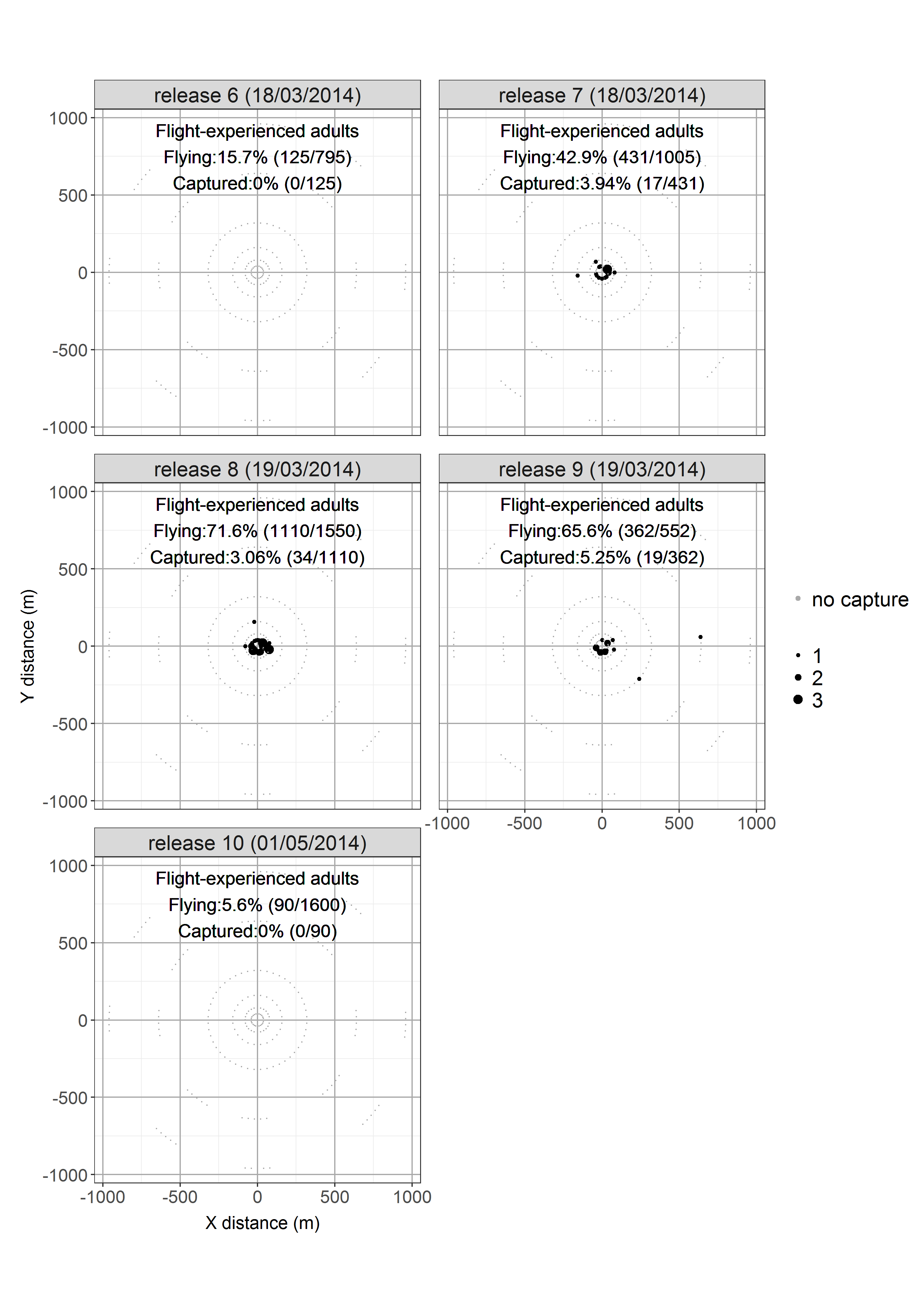
**

**Fig B. Spatial location and number of marked *Hylurgus ligniperda* individuals recaptured in releases 6 to 10.**

**
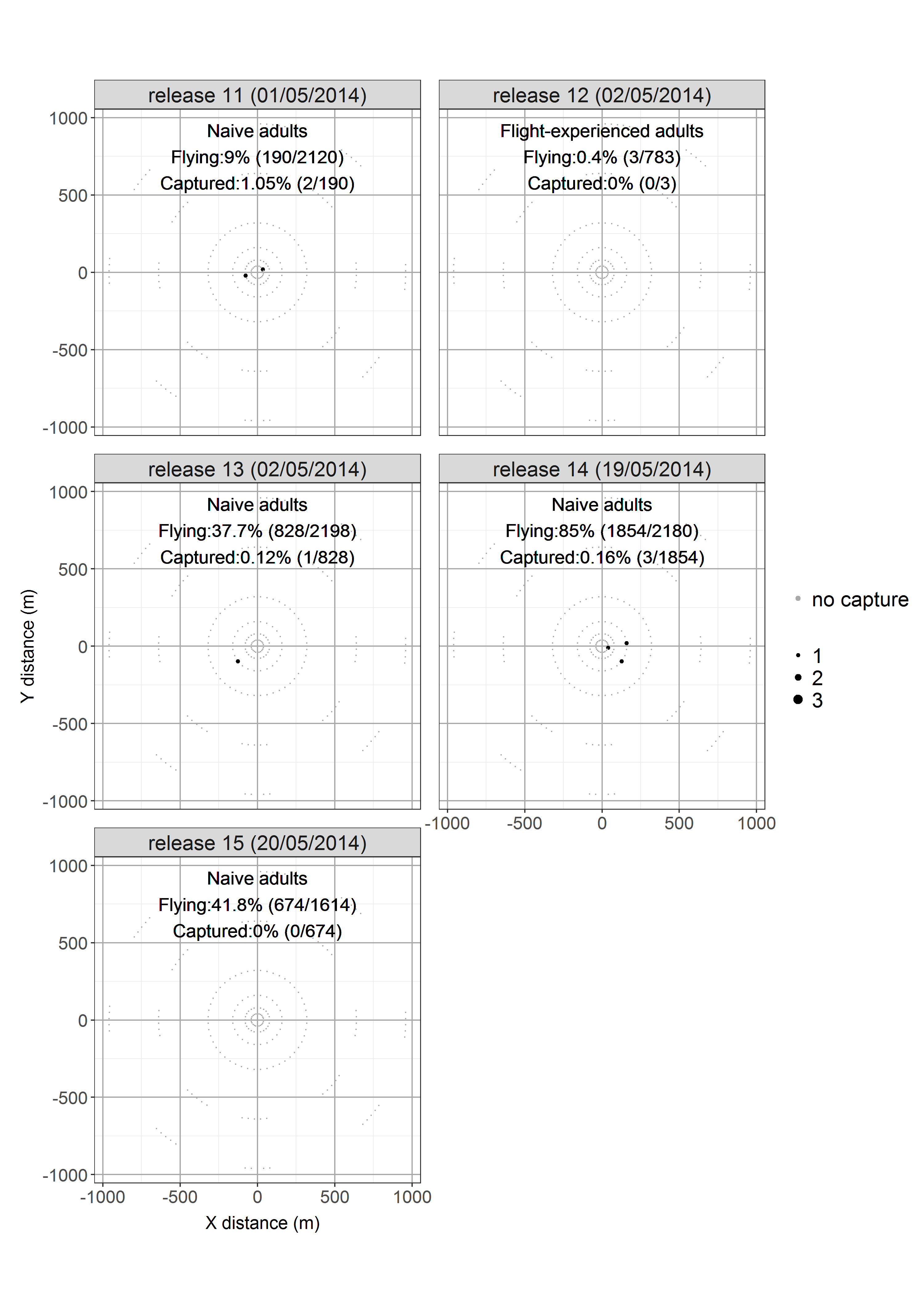
**

**Fig C. Spatial location and number of marked *Hylurgus ligniperda* individuals recaptured in releases 11 to 15.**

**
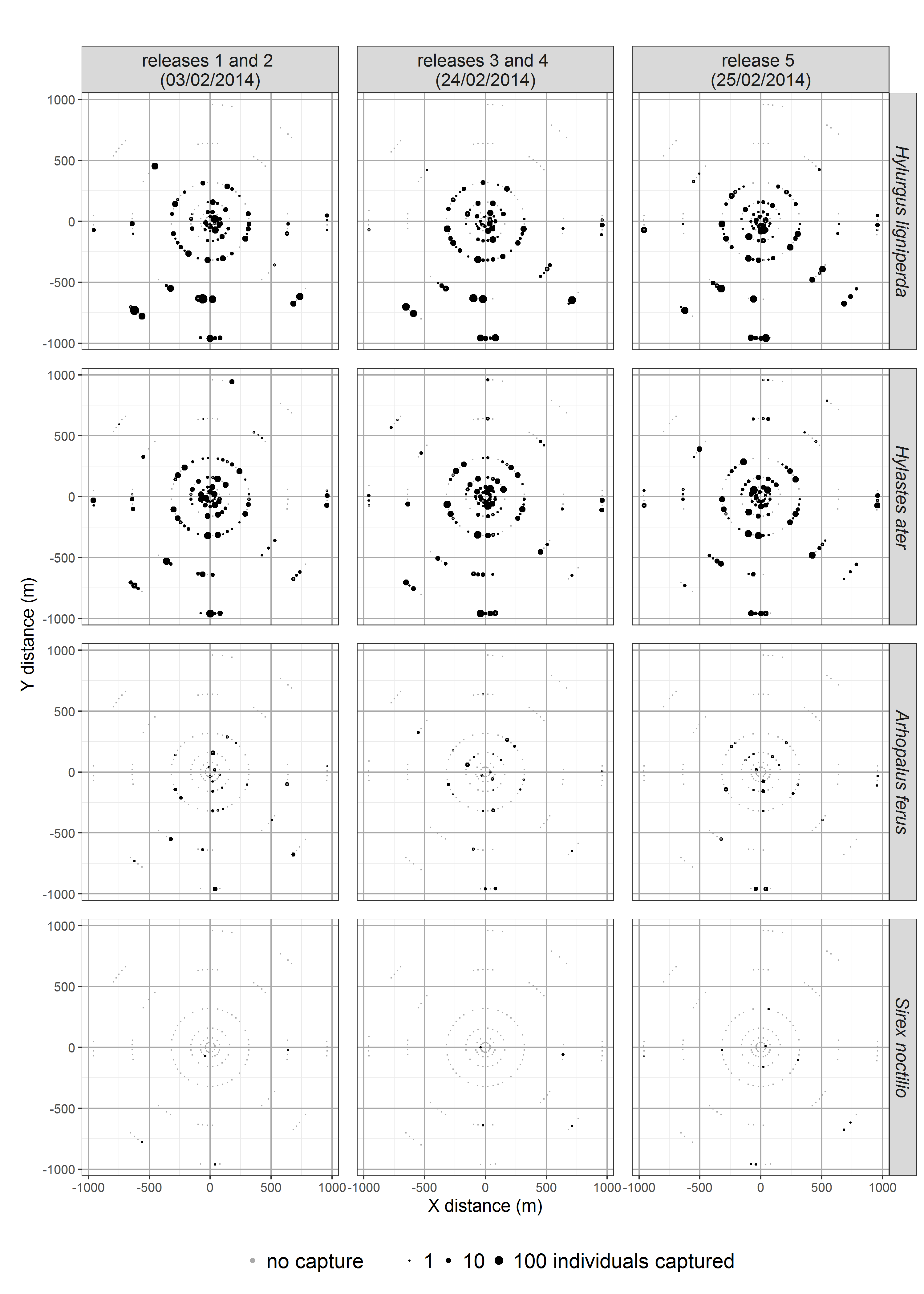
Fig D. Spatial location and number of wild *Hylurgus ligniperda*, *Hylaster ater*, *Arhopalus ferus* and *Sirex noctilio* captured in releases 1 to 5.**

**
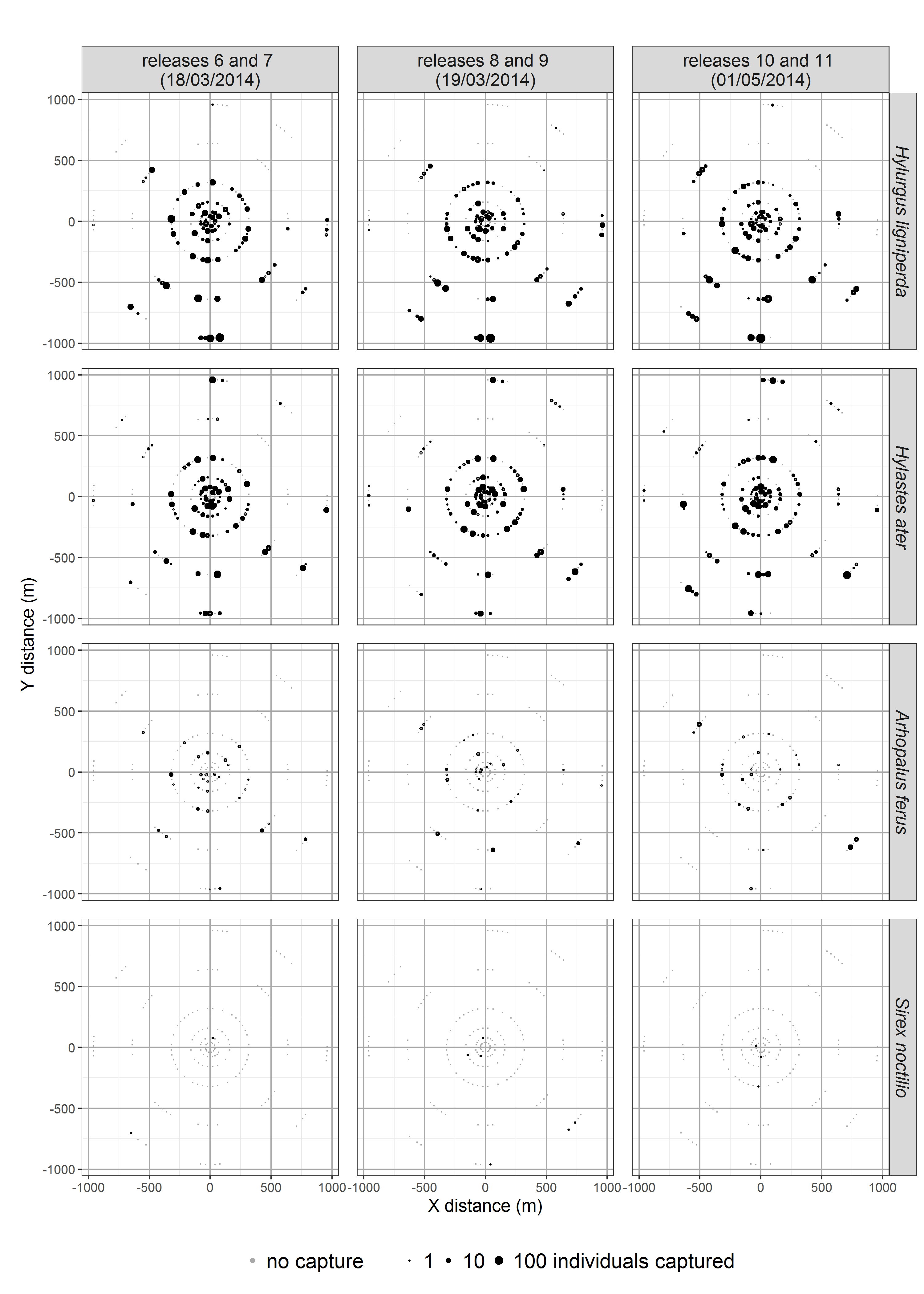
Fig E. Spatial location and number of wild *Hylurgus ligniperda*, *Hylaster ater*, *Arhopalus ferus* and *Sirex noctilio* captured in releases 6 to 11.**

**
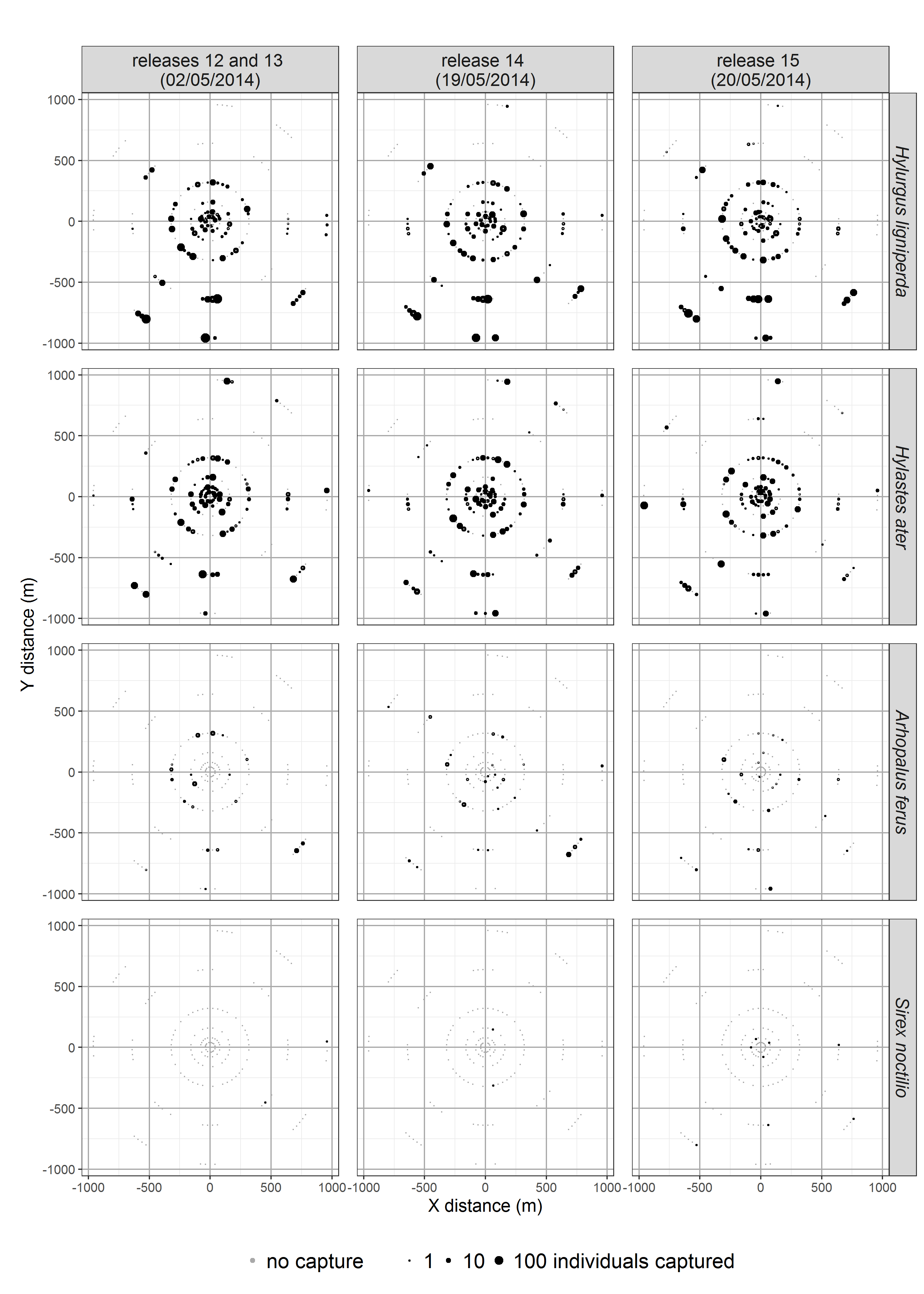
Fig F. Spatial location and number of wild *Hylurgus ligniperda*, *Hylaster ater*, *Arhopalus ferus* and *Sirex noctilio* captured in releases 12 to 15.**
